# Advancing brain barriers RNA sequencing: guidelines from experimental design to publication

**DOI:** 10.1186/s12987-020-00207-2

**Published:** 2020-08-18

**Authors:** David M. F. Francisco, Luca Marchetti, Sabela Rodríguez-Lorenzo, Eduardo Frías-Anaya, Ricardo M. Figueiredo, BtRAIN Network, Marjolein Heymanns, Maxime Culot, Ana Raquel Santa-Maria, Maria A. Deli, Raoul F. V. Germano, Benoit Vanhollebeke, Nikolaos Kakogiannos, Monica Giannotta, Elisabetta Dejana, Amaia Dominguez-Belloso, Stefan Liebner, Markus Schuster, Harm-Anton Klok, Marie Wiatr, Horst Schroten, Tobias Tenenbaum, Gijs Kooij, Peter Winter, Ignacio Andres Romero, Helga E. de Vries, Britta Engelhardt, Rémy Bruggmann

**Affiliations:** 1 Interfaculty Bioinformatics Unit and Swiss Institute of Bioinformatics, University of Bern, Bern, Switzerland; 2 Theodor Kocher Institute, University of Bern, Bern, Switzerland; 3 MS Center Amsterdam, Amsterdam Neuroscience, Department of Molecular Cell Biology and Immunology, Amsterdam UMC, Vrije Universiteit Amsterdam, Amsterdam, The Netherlands; 4 School of Life, Health and Chemical Sciences, The Open University, Milton Keynes, UK; 5 GenXPro GmbH, Frankfurt/Main, Germany; 6 Johann Wolfgang Goethe University, Frankfurt/Main, Germany

## Abstract

**Background:**

RNA sequencing (RNA-Seq) in its varied forms has become an indispensable tool for analyzing differential gene expression and thus characterization of specific tissues. Aiming to understand the brain barriers genetic signature, RNA seq has also been introduced in brain barriers research. This has led to availability of both, bulk and single-cell RNA-Seq datasets over the last few years. If appropriately performed, the RNA-Seq studies provide powerful datasets that allow for significant deepening of knowledge on the molecular mechanisms that establish the brain barriers. However, RNA-Seq studies comprise complex workflows that require to consider many options and variables before, during and after the proper sequencing process.

**Main body:**

In the current manuscript, we build on the interdisciplinary experience of the European PhD Training Network BtRAIN (https://www.btrain-2020.eu/) where bioinformaticians and brain barriers researchers collaborated to analyze and establish RNA-Seq datasets on vertebrate brain barriers. The obstacles BtRAIN has identified in this process have been integrated into the present manuscript. It provides guidelines along the entire workflow of brain barriers RNA-Seq studies starting from the overall experimental design to interpretation of results. Focusing on the vertebrate endothelial blood–brain barrier (BBB) and epithelial blood-cerebrospinal-fluid barrier (BCSFB) of the choroid plexus, we provide a step-by-step description of the workflow, highlighting the decisions to be made at each step of the workflow and explaining the strengths and weaknesses of individual choices made. Finally, we propose recommendations for accurate data interpretation and on the information to be included into a publication to ensure appropriate accessibility of the data and reproducibility of the observations by the scientific community.

**Conclusion:**

Next generation transcriptomic profiling of the brain barriers provides a novel resource for understanding the development, function and pathology of these barrier cells, which is essential for understanding CNS homeostasis and disease. Continuous advancement and sophistication of RNA-Seq will require interdisciplinary approaches between brain barrier researchers and bioinformaticians as successfully performed in BtRAIN. The present guidelines are built on the BtRAIN interdisciplinary experience and aim to facilitate collaboration of brain barriers researchers with bioinformaticians to advance RNA-Seq study design in the brain barriers community.

## Background

### Brain barriers: terms and definitions

Central nervous system (CNS) homeostasis is ensured by endothelial, epithelial, mesothelial and glial brain barriers that divide the CNS into compartments [1]. CNS barriers allow undisturbed neuronal function within the parenchyma while ensuring immune surveillance at the borders of the CNS.

**Fig. 1:**
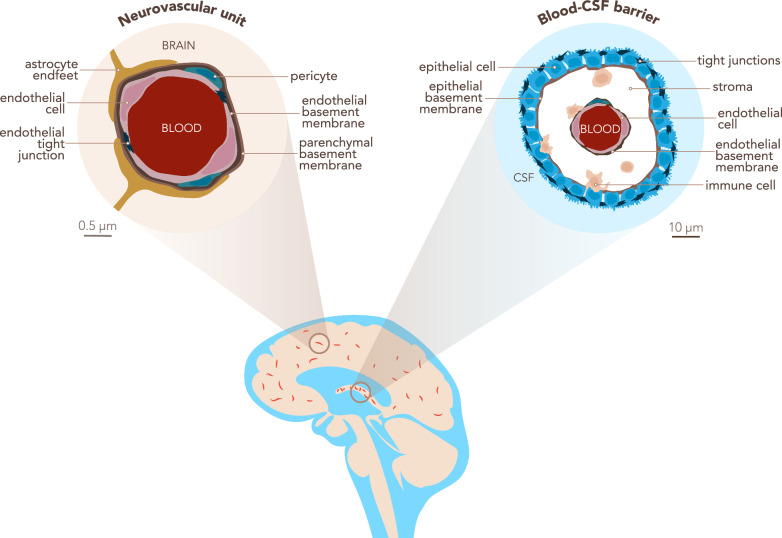
The blood–brain barrier in the context of the neurovascular unit and the blood-CSF barrier. The blood–brain barrier (BBB) is located within the neurovascular unit (NVU, left scheme) at the level of the brain parenchymal microvasculature and composed of *endothelial cells* tightly connected by unique *tight junctions*. It separates brain *parenchyma* from the peripheral *blood*. Endothelial cells produce a basement membrane in which *pericytes* are embedded. *Astrocyte endfeet* closely contact the microvessels and astrocytes lay down the *parenchymal basement membrane*. The choroid plexus (ChP) stroma is separated from the CSF space by the blood-CSF barrier (BCSFB, right scheme), which is composed of ChP *epithelial cells* tightly connected by apical *tight junctions*. The apical side of the epithelium faces the *CSF*, while the basolateral side resting on an *epithelial basement membrane* faces the ChP stroma. The ChP stroma is highly vascularized with *blood* vessels lacking a BBB and populated by *immune cells*. The *endothelial cells* produce their own *endothelial basement membrane*

For the purpose of clarity, we here define some general terms, as they lack a cohesive reference within the brain barriers community. For the purposes of this manuscript: The blood–brain barrier (BBB) is localized at the level of endothelial cells of the CNS microvasculature, which includes capillaries, pre-capillary arterioles and post-capillaries venules. BBB characteristics are not intrinsic to CNS microvascular endothelial cells but rather rely on the continuous crosstalk of cellular and acellular elements around CNS microvessels, which are referred to as the neurovascular unit (NVU). The NVU contains BBB endothelial cells, the endothelial basement membrane with a high number of embedded pericytes and the glia limitans composed of the parenchymal basement membrane and astrocytic endfeet [2]. The blood-cerebrospinal fluid barrier (BCSFB) is composed of epithelial cells surrounding the choroid plexuses (ChP), which extend into the cerebrospinal fluid (CSF) filled brain ventricles (Fig. 1).

The known functions of the BBB and BCSFB include inhibition of free diffusion of molecules from the blood to the CNS while ensuring rapid efflux of toxic metabolites out of the CNS [3]. In addition, both the BBB and BCSFB control immune cell entry into the CNS [4–6]. The present study does not include references to the following CNS barriers: The arachnoid mater, which establishes a BCSFB between the dura mater lacking a BBB and the CSF filled subarachnoid space [7]. The pia mater, which is localized at the surface of the brain and spinal cord and embraces the subarachnoid arteries [4]. The glia limitans, which ensheaths the entire CNS parenchyma [4].

### Endothelial cells of the BBB are biochemically unique

The BBB endothelial cells are characterized by the presence of molecularly unique, complex and continuous tight junctions, in addition to adherens junctions, lack of fenestrations and a low rate of pinocytotic activity [8, 9]. Moreover, BBB endothelial cells express specific enzymes and transporters that allow an efficient transport of nutrients into the CNS and efflux of toxic metabolites out of the CNS [10, 11]. Despite these unique biochemical characteristics, endothelial cells of the BBB share some properties with endothelial cells in peripheral microvascular beds. For example, all endothelial cells develop adherens junctions and may express tight junction proteins, but at the BBB, adherens junctions are accompanied by complex and continuous tight junction strands surrounding the entire circumference of the brain microvascular endothelial cells [1]. A better understanding of the unique structural and functional characteristics of the BBB endothelium would significantly improve our understanding of the contribution of BBB impairment in neurological disorders.

Phenotypic characteristics of the brain barriers are ultimately regulated at the transcriptional level. In fact, the analysis of transcription profiles has been a useful tool in biomedical research and has had an increasing impact in the last few decades. Therefore, different research groups have begun to investigate the BBB transcriptome employing different methodologies. For instance, the first RNA-Seq study of CNS cells, including the BBB, was published 10 years ago [12] while a recent study made use of claudin-5-GFP reporter mice to sort GFP^+^ endothelial cells from the brain of mice for subsequent single cell RNAseq (scRNA-Seq) [13]. This study identified zonated transcriptional profiles of brain endothelial cells along the arteriovenous axis [13]. Other studies have employed bulk RNA-Seq of endothelial cells isolated from the brain and peripheral tissues of VE-Cadherin-Cre^ERT2^-Rosa-tdTomato mice to isolate endothelial cells from the brain and peripheral tissues in health and disease, including mouse models of stroke, multiple sclerosis, traumatic brain injury and seizures [14]. This molecular profiling has defined core BBB genes expressed by brain endothelial cells that become deregulated in pathology, suggesting potential therapeutic targets common to multiple neurological disorders [14]. These approaches underscore the relevance of transcriptomic profiling of the brain barrier cells to advance our understanding of the molecular pathways underlying brain barriers function and dysfunction.

### Progression of transcriptional analysis techniques: from Sanger to next generation sequencing

Throughout the 1980s, Sanger sequencing was used to identify transcripts within tissues and cells, while quantitative methodologies, such as quantitative real-time polymerase chain reaction (qRT-PCR), came into prominence in the 1990s. These methods, usually referred to as low-throughput or ‘first-generation’ sequencing, are still being used to this day for specific purposes, although they are laborious, costly (for output level) and therefore, not suitable for establishing full transcriptomes of an entire tissue. The new millennium brought high-throughput techniques for transcriptomics analysis, with microarrays followed by next-generation sequencing (NGS) technologies. The most relevant in the present context and the most commonly used NGS technique is RNA-Seq, which allows advances in the characterization and quantification of transcriptomes, including whole transcriptome sequencing in a much less laborious and time-consuming fashion compared to previous methods [15–17].

**Fig. 2:**
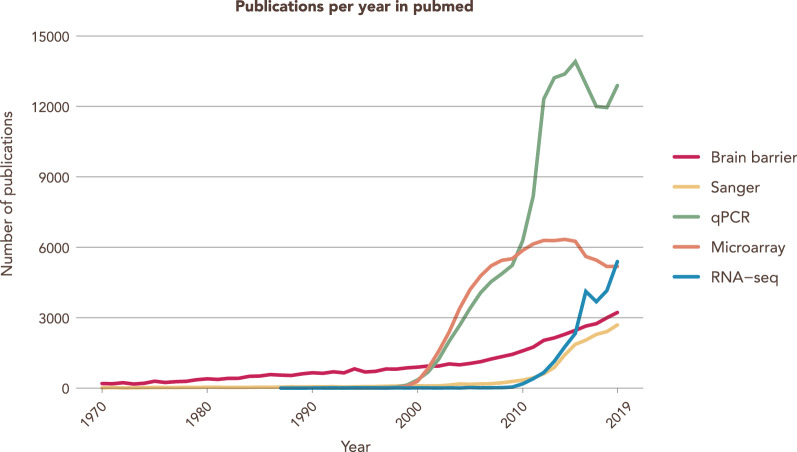
RNA-Seq is an increasingly popular tool for transcriptional analysis. Number of searchable publications in Pubmed per year by the terms specified in the legend. Data source: Pubmed. Search date 31/12/2019. Y-axis shows number of publications, X-axis shows years

New technologies bring along opportunities that allow for a more in depth understanding of known mechanisms and the discovery of novel pathways. The new challenges and problems must be addressed, and so, the field of bioinformatics with its associated researchers who are highly specialized data analysts, started. In their short history, RNA-Seq methods have seen a sharp decline in costs coupled with the improvement of the underlying technology. This translated to an exponential increase of studies and groups taking advantage of this technology and of the amount of large datasets produced and published (Fig. 2).

### Transcriptome analysis and brain barriers: challenges and manuscript objectives

A major challenge when aiming to compare transcriptome profiles from a given cell is understanding the source of the cell and how it was isolated. In the present context it is important to highlight that we found discrepancies in the protocols used for isolating brain endothelial cells to be common but remarkably underreported. The inaccuracy begins with a lack of consensus in the nomenclature for the different CNS vascular segments isolated and analyzed, with some laboratories referring to *brain microvessels* when isolating pure capillary fractions and others referring to *capillaries* when in fact the isolated microvessels are comprised of a mixture of arterioles, venules and capillaries. Considering the reported zonated gene expression of endothelial cells along the CNS vascular tree [13], transcriptome profiling studies performed on the BBB can hardly be compared, as most of the published studies lack an in depth description of the CNS endothelial isolation procedures.

To unveil the full power of transcriptome profiling it is, thus, essential to have a solid intersection in the fields of transcriptome profiling, bioinformatic analysis and classical brain barriers research. In this manuscript we highlight the intersection of transcriptomic profiling (with an emphasis on RNA-Seq) and the field of studying the brain barriers (with an emphasis on the endothelial BBB and the epithelial BCSFB). We start by addressing considerations to be taken into account for the overall experimental design, and then elaborate on the multiple and essential intermediate steps throughout the workflow, including comparing different BBB isolation methodologies for RNA-Seq, data analysis and publishing recommendations. It is not our intention to establish rigid rules on how to perform an RNA-seq study in the field of brain barriers. Rather, our aim, based on our collaborative approaches in BtRAIN, is to raise awareness of the relevance of each experimental step and to highlight the relative strengths and weaknesses of the available alternatives. We then summarize what we consider essential information to be included in original manuscripts describing RNA-seq to define BBB signature genes in health and disease. We are convinced that appropriate availability of information will improve comparability and reproducibility of the different studies and thus advance quality and cost-efficiency of these studies in the field. By setting the stage for datasets that allow for meta-analysis-based research, our suggestions will furthermore allow for the implementation of the 3R rules of experimental animal research by reducing and refining animal experimentation.

## Main body

### Considerations for the experimental design of a BBB RNA-Seq study

Experimental design is possibly the most important step of any transcriptomic experiment as the success of the project heavily depends on the choices made at this early stage. The first step is to have clear and defined goals. Questions that should be addressed before starting an experiment include: (i) Is the intent of the experiment to specifically define the transcriptome of the brain endothelial cells along the entire vascular tree or rather solely of BBB endothelial cells in CNS microvessels or even capillaries? (ii) Is the aim to compare the transcriptome of brain endothelial cells at different stages of e.g. development or under specific pathological conditions? (iii) Is the intent to define the transcriptome of a specific tissue (i.e. ChP epithelial cells vs kidney epithelial cells), a specific time point (i.e. embryonic vs post-natal BBB development) or a specific pathological condition (Multiple Sclerosis vs Alzheimer’s Disease)? (iv) What are the possible batch effects, such as the sacrificing groups of experimental animals on different days? These, and other similar questions, are known as intrinsic factors and their impact on the experimental design is direct, as they are defined by the question and objectives of the study.

**Fig. 3:**
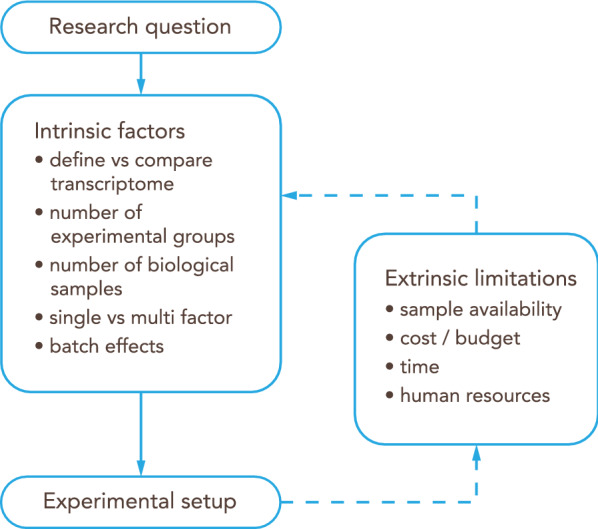
Workflow for the experimental design of an RNA-Seq study. The research question will guide the initial experimental setup based on intrinsic factors. Then, extrinsic limitations should be taken into account to adjust and refine the overall design

There are also extrinsic factors that influence experimental design in the form of practical limitations. They are (i) biological sample availability, e.g.: human CNS tissue is sparse and may not be obtained in the required quality to allow for RNAseq analysis. (ii) costs, e.g.: pre-sequencing optimization costs as well as sufficient sequencing of samples per group to the required depth. (iii) time, e.g. time required for breeding experimental animals to obtain the required brain barriers genotype, time for protocol optimization of the tissue of interest (be it BBB, NVU, whole cortex or others) isolation protocol and validation of the results and iv) human resources, as a transcriptomics project might involve several scientists, from the principal investigator to wet lab researchers and technicians, sequencing facility technicians and bioinformaticians (Fig. 3).

#### Guidelines

- Define a clear goal for the transcriptomics study: What is the specific intention for the project.
- Consider both extrinsic and intrinsic factors when designing an experiment.
- Plan the experiment including the advice from the experts involved in the different steps, such as sequencing facility staff and bioinformaticians.

### Vascular heterogeneity in the CNS to be considered when characterizing the BBB transcriptome

**Fig. 4:**
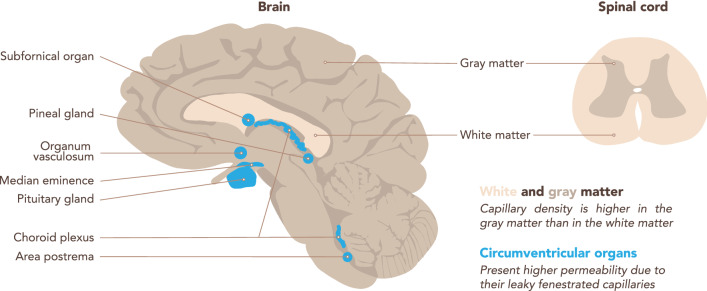
Regional differences in the brain microvasculature. Schematic representation of a brain sagittal section (left) and a spinal cord transverse section (right). Capillary density is higher in the CNS gray matter than in the white matter, according to their differential metabolic activity. The white matter of the corpus callosum is highlighted. The microvessels in the circumventricular organs (CVOs, highlighted in blue) lack BBB characteristics, rather they are fenestrated and thus permeable to blood components. CVOs include the subfornical organ, the vascular organ of the lamina terminalis, the area postrema, the median eminence, the neurohypophysis, the pineal gland, and the choroid plexus

The vasculature is heterogeneous throughout the CNS [18] (Fig. 4). This heterogeneity is reflected in the transcriptome, and therefore should be considered prior to isolating CNS microvessels for a transcriptomic study of the BBB. Two main factors are the capillary density and the BBB properties, which may in addition be affected by age, sex or the pathological conditions investigated.

Capillary density is related to the metabolic demands, and thus neuronal activity, of the respective CNS regions [19]. The gray matter (GM) of the cerebral cortex harbors many neuronal cell bodies and is therefore more metabolically active than the white matter (WM), where the myelinated axonal fibers run. Thus, GM harbors a higher density of capillaries when compared to WM [20]. In addition, there are specific regions in the CNS, such as the hippocampus, characterized by a remarkable heterogeneity in capillary density [21].

The cellular and molecular characteristics of the NVU components are also heterogeneous throughout the CNS. Endothelial cells of the BBB present some of the highest regional differences. Indeed, expression of endothelial junction proteins (occludin, claudin-5 and a-catenin) is higher in the WM compared to the GM [22]. In the blood spinal cord barrier, the endothelium is less tight and is characterized by a lower pericyte coverage [18]. Astrocytes also show heterogeneity along the brain vasculature, including higher expression of glial fibrillary acidic protein (GFAP), an intermediate filament, in WM relative to GM [22, 23]. In contrast, the expression of aquaporin-4 (AQP4), a water channel localized at astrocyte endfeet, is more homogenous throughout the perivascular glia limitans [22, 24]. Moreover, endothelial cells do not form a BBB throughout the whole CNS. Particularly microvessels within the circumventricular organs (CVOs) lack BBB properties. CVOs are localized around the brain ventricles and fulfill neurosecretory and neurosensory functions. The CVOs include the subfornical organ, the vascular organ of the lamina terminalis, the area postrema, the median eminence, the neurohypophysis, the pineal gland, and also the ChPs (Fig. 4). CVOs contain fenestrated microvessels that allow for the free diffusion of blood components into the CVO stroma. Thus, co-isolation of microvessels from the CVOs should be avoided when aiming to specifically analyze the BBB transcriptome [25].

**Fig. 5:**
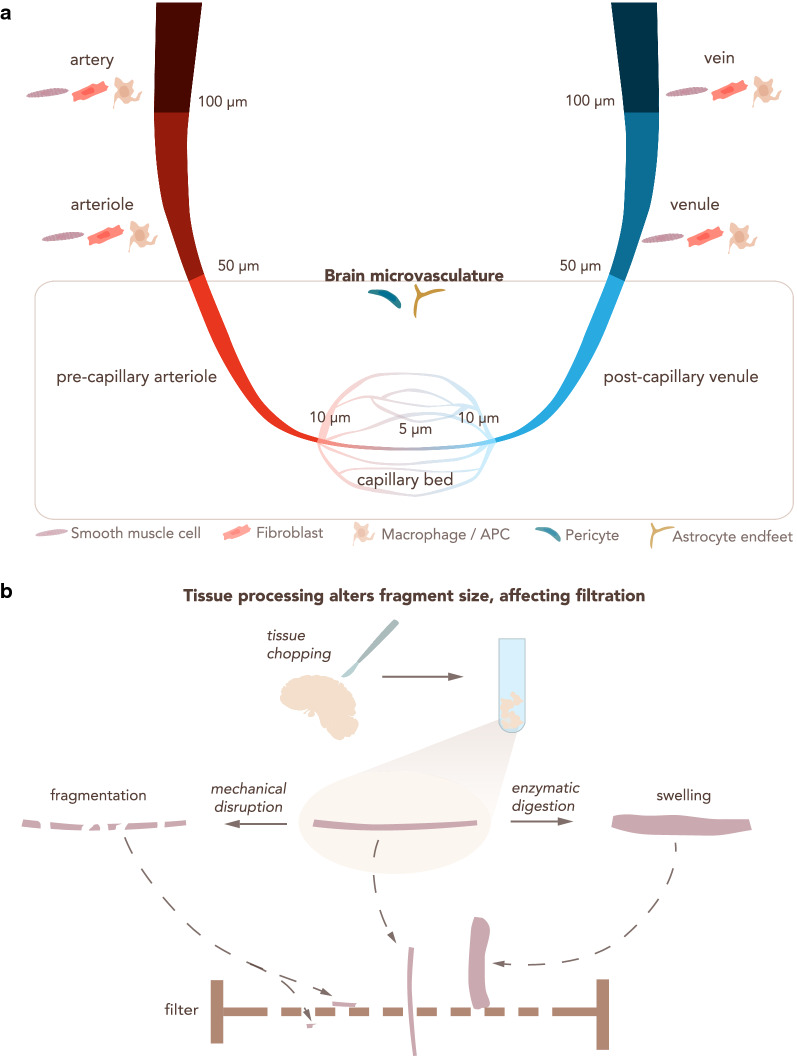
Heterogeneity in microvasculature diameter determines the outcome of filtration steps for BBB enrichment. **a** The CNS vascular tree from arteries to veins. An indicative range of vessel diameter for each vascular section is provided, along with other cell types that may be co-isolated. Arteries and veins have a diameter > 100 μm, arterioles and venules from 100 μm to 50 μm. The brain microvasculature has a diameter smaller than 50 μm and consists of pre-capillary arterioles, capillaries and post-capillary venules. Capillaries are generally considered those with a diameter < 10 μm and often show diameters of about 5 μm. APC: Antigen-presenting cell. **b** Enzymatic digestion and mechanical disruption during CNS vascular isolation protocols alter the physical properties of the microvasculature fragments, influencing the downstream steps of the isolation protocol, particularly size dependent filtration across nylon membranes. Small vascular fragments obtained by mechanical disruption can allow for undesired vessels to pass through the filter. Enzymatic digestion leads to swelling of the blood vessels, prohibiting their elution through the filter

The vascular tree presents gradual phenotypic heterogeneity, a phenomenon known as zonation, accompanied by transcriptional differences [13]. Organization of TJs, rate of pinocytosis, expression of enzymes such as alkaline phosphatase, Na^+^/K^+^ ATPase, expression of transporters or efflux pumps or of adhesion molecules are not the same in endothelial cells of brain arterioles, capillaries and venules (Table 1) [26], in line with the different functions of these vascular segments. In addition, the mural cell subsets in these microvascular segments differ, with smooth muscle cells embracing arterioles and, to a lesser degree, venules, while pericytes are highly concentrated at the level of the capillaries [27, 28] (Fig. 5).

**Table 1:** Segmental heterogeneity in the brain microvasculature endothelial cells (arterioles, capillaries and venules). Adapted from [29] and [30]

	Arterioles	Capillaries	References
Cells			
Smooth muscle cells	Yes	No	[13, 30]
Pericytes	Yes/No	Yes	[13, 31]
Perivascular macrophages	Yes	Yes	[13, 30]
BBB features			
Tight junctions (TJs)	Continuous and elaborate TJs/?	Highly expressed TJs	[29, 32, 33]
Permeability for BBB markers	?	No	[30, 34]
Astrocytic end-foot shealth	No	Yes	[29, 30]
Transporters/enzymes/receptors			
P-glycoprotein	?	+++	[35, 36]
Na^+^/K^+^-ATPase	+++	+	[29]
Transferrin receptor (TFRC)	–	+++	[13, 37]
Alkaline phosphatase	+++	+++	[29]
Mg^2+^-ATPase	+++	+	[29]
5′-nucleotidase	+++	+	[29]
γ-Glutamyl transpeptidase (GGTP)	?	+++	[38, 39]
Bidirectiona/l vesicular horsedish peroxidase transport	+++	+	[29, 40]

Presence of perivascular cells is indicated by Yes/No. Presence of BBB features is indicated by Yes/No/non determined (n.d.). Relative expression of transporters/enzymes is indicated by +, ++ or +++ from lowest to highest expression

Vascular heterogeneity can also be induced by CNS pathology, e.g. neuroinflammation, neurodegeneration or brain tumors, which may in fact lead to focal alterations in the NVU associated with the CNS pathology. This may range from changes in cellular composition of the NVU, e.g. pericyte drop during stroke, perivascular accumulation of inflammatory cells in multiple sclerosis or to alterations in vessel diameters as observed in brain tumors. All these alterations will affect the outcome of established brain barrier cell isolation protocols with respect to purity of the brain barrier cells as well as RNA stability.

#### Guidelines

- Consider CNS capillary density of the region of interest to obtain enough RNA yield: capillary density is higher in GM compared to WM.
- Consider regional differences in BBB properties. Clearly indicate from which CNS region microvessels, capillaries or endothelial cells were isolated.
- Consider if you wish to analyze the transcriptome of endothelial cells from a specific vascular segment or a specific region of the CNS.
- Consider general or regional alterations in BBB properties when studying CNS pathologies. Clearly indicate from which CNS region and at what disease stage the microvessels, capillaries or endothelial cells were isolated.

### Regional heterogeneity among the four choroid plexus to consider for analyzing the BCSFB transcriptome

The choroid plexus (ChPs) protrude into each of the brain ventricles, and thus there are two lateral (telencephalic) ChPs, one in the third ventricle (diencephalic) and one in the fourth ventricle (hindbrain or myelencephalic). There is increasing evidence that the gene expression profile of each ChP reflects their positional identities. The mouse lateral and fourth ventricle whole ChPs present a differential transcriptome and secretome, as assessed by RNA-seq and mass spectrometry [41]. A recent single nucleus RNA-seq study revealed a unique cellular composition in each of the mouse ChPs [42]. Of note, the more regionalized cell types were epithelial cells and fibroblasts, while ChP endothelial cells were found to be more homogeneous across the ventricles.

Although most transcriptomic studies have focused on the lateral ChPs, the choice of ChP will influence the sequencing results and the comparison with available datasets. The heterogeneity of the ChP among the four ventricles in the human brain remains to be characterized.

#### Guidelines

- Consider regional transcriptional heterogeneity among the four ChPs. The choice of ChP should be stated in the methods.

### Brief recommendations on how to select a reporter mouse

Many genetically modified mouse models have been developed for studying brain barriers development and function. For a general overview on the available genetic tools that are used to study the BBB function, we recommend the review of Sohet et al. [43], while for a study involving a solid report in the BCSFB we recommend the reading of Johnson et al. [44]. In the context of this manuscript the brain barriers reporter mouse lines that allow to distinguish brain barriers endothelial or epithelial cells from other cells of the CNS by means of expression of a fluorescent reporter are of specific interest. While many of those mouse lines have been developed for imaging purposes they also allow for sorting of the cells of interest from the CNS based on expression of their fluorescent reporter [12, 13, 45]. For the purpose of this manuscript, we will simply mention some recommendations and possible pitfalls while using genetic mouse models in RNA-Seq studies in the brain barriers field.

Before any experimental approach, a deep understanding of the genetic mouse model that is used is needed. To this end, online tools such as http://www.informatics.jax.org/ can be used to have a detailed overview of the mouse line of interest. Original literature on how the mouse line has been created and the expression pattern of the respective reporter needs to be carefully evaluated. This includes consideration of the promoter used for driving the expression of the fluorescent reporter with respect to cellular specificity or efficiency of expression, which could depend also on the age or disease state investigated. Furthermore, inducible expression systems, e.g. based on Cre-recombinase or TET-ON or TET-OFF-regulation need to be tested for their specificity, leakiness and completeness of driving reporter expression [46, 47]. Specificity and intensity of the brain barriers reporter expression should always be tested in house prior to using the respective mouse model for sorting of brain barriers cells.

## Considerations for protocols on the isolation of CNS microvessels, capillaries or single endothelial cells

A RNA-Seq study of CNS microvessels, capillaries or single BBB endothelial cells necessarily relies on the protocol used to isolate the target tissue or cell. Due to the highly complex structure of the NVU and the aforementioned heterogeneity of NVU components throughout the CNS vasculature, a detailed description of the protocol used to isolate the material for RNA-seq analysis is mandatory. Each step of the isolation protocol is critical and has a direct impact on the sample purity as well as on the yield of the material obtained for sequencing. To date, many different protocols for the isolation of endothelial cells of the BBB, or CNS microvessels, or brain endothelial cells have been published. Most classical isolation protocols consist of a combination of mechanical disruption and enzymatic digestion and a subsequent size selection of the isolated vascular segments by filtration through nylon membranes with different pore sizes (Fig. 5). Additional enrichment of the respective vascular segment is usually achieved by density gradient separation [48–50]. More refined techniques can be incorporated to increase purity, such as selection with antibody-coupled magnetic beads or FACS, or taking advantage of fluorescent reporter mice [51], as discussed above.

## BBB microvessel or capillary isolation

### Tissue disaggregation: enzymatic digestion versus mechanical disruption

The first step for the isolation of CNS microvessels or endothelial cells of the BBB is to properly slice or cut the tissue into small pieces in order to facilitate enzyme-based digestion or the following Dounce homogenization steps [52]. Typical techniques of brain tissue disaggregation are either enzymatic or mechanical. Enzymatic digestion is often performed using a combination of enzymes such as collagenase and dispase as well as DNAse. Mechanical disruption, on the other hand, is usually performed using Dounce homogenizers with different loose pestle sizes depending on the amount of tissue and the selected species [53, 54]. This ensures tissue loosening by shear forces without affecting cell viability. Mechanical techniques may prove to be more effective while at the same time may be too harsh depending on certain factors, such as CNS region or age of the individual (i.e. aged tissue is more susceptible to damage). Therefore, the choice of technique for tissue disaggregation can influence the rest of the protocol and should be described in detail in the methods of the study.

### Microvessel hierarchy selection depending on size: filtration steps

Filtration of the dissociated tissue or CNS vasculature fragments enriches for a specific component from the brain homogenate. One of the most common methods for the isolation of CNS microvessels or specifically capillaries is performing either single or a series of filtrations through nylon or polyester membranes followed by gradient centrifugation steps with Percoll™, Dextran or serum albumin, in order to separate microvessel fragments from cellular debris, myelin and other non-desired cell types [55–58].

The enrichment of a certain type of CNS microvessel (i.e. capillaries) over the others depends on their diameter and can be achieved by using different filter pore sizes. Therefore, the combination of larger (~ 100 μm) to smaller (~ 20 μm) filter pore sizes, in addition to using one or several filters in sequence, can determine the final vascular segment that is isolated [59]. Indeed, the choice of meshes should take into account the different vessel caliber of the CNS vasculature tree (Fig. 5a). Generally speaking, arteries have a diameter ≥ 100 μm, arterioles and venules between 100 and 50 μm, post-capillary venules and pre-capillary arterioles between 50 and 10 μm, and capillaries are considered to have a diameter ≤ 10 μm [60–62]. The diameter of the arteries decreases from the surface of the brain towards the deeper regions [63]. Moreover, variability in brain artery diameter between different mouse strains has been observed [64], underscoring the necessity of a detailed description of the source of sequencing material. In humans, CNS vessel diameters are affected by the health status of the donor [65] while in rat age was shown to contribute to reduced capillary diameter in the brain stem [66].

Although size selection represents a possible choice for defining the CNS microvascular segment that is isolated, some technical details have to be kept in mind. Other steps of the protocol can highly influence the physical properties of the isolated microvessels or capillaries (Fig. 5b). For instance, mechanical disruption reduces the length of the vasculature fragments and therefore might impact size selection. Enzymatic digestion causes swelling of the microvasculature fragments, increasing their diameter. Due to these technical aspects, the researcher should use the size filtration as a guideline, and empirically determine the exact fraction of the vascular tree that has been purified at the end of the isolation protocol (visually analyzing the vascular fragment). Alternate tools that help achieve this validation include publicly available scRNA-Seq datasets of different BBB cell types [13]. These datasets collect information about specific cell markers that can be used to complement the isolation. However, it should be clear that expression of a few chosen markers might not successfully identify specific microvascular segments and that gene expression differences along brain endothelial cells conform to a gradient rather than discrete segments.

In sum, size selection is a critical step in the isolation protocol of microvessels, capillaries or individual endothelial cells of the BBB, and ambiguity in terminology should be avoided by accompanying qualitative and quantitative information.

#### Guidelines

- Tissue processing methods can affect size selection. Indicate the method used and, if possible, the state of the tissue after dissociation.
- Indicate the size-dependent filtration steps, if used, including pore size of the filter mesh and the combination of more than one filter.
- For animal studies, indicate the strain, age and sex of the animals used. For reporter mouse studies indicate the precise mouse line used as indicated on the MGI homepage (http://www.informatics.jax.org).
- For human studies, provide age, sex and relevant clinical information of the individuals.

## Isolation methods of microvessel fragments or single endothelial cells from the BBB

### Strategies for BBB purity refinement: selection with antibody-coupled magnetic beads

Selection via magnetic beads coated with specific antibodies represents a useful and precise method to isolate microvessels or endothelial cells of the BBB. This technique can be used for positive selection of the material of interest or for negative selection of possible contaminants, alone or combined. For example, to purify endothelial cells, positive selection can be achieved by using beads coated with anti-CD31 and/or VE-cadherin antibodies [67, 68]. On the other hand, beads coated with antibodies against CD68, PDGFRβ, NG2 or GLAST might be used to specifically select macrophages, pericytes and astrocytes respectively [69–73], or to deplete these cells in those cases where a pure endothelial fraction is required. Positive and negative selection may be combined in order to improve the specificity of the technique.

Despite the high selectivity and improved final purity that the bead-mediated selection offers by targeting specific cell types, some disadvantages need to be considered. First, the state of the vessel suspension and the physical interaction between different BBB components is a critical factor. During the isolation protocol, the brain vessels are not revealed in a single cell suspension but rather as vascular fragments that consist of tightly connected cell types, such as endothelial cells, pericytes and astrocyte endfeet [59]. Prior to bead selection, additional disaggregation steps, including enzymatic or mechanical disruption, might aid in obtaining a higher fraction of single cells versus microvessel fragments by weakening the interaction between different cell types, which may enhance the disassociation and determine the outcome of the isolation [54]. The close interaction between the different components of the BBB [74, 75], makes a total single cell suspension from CNS microvessels a challenging task, ultimately limiting the availability of binding sites for the antibodies and influencing the isolation efficiency if not performed properly. An additional aspect to consider is that the enzymatic digestion may influence the surface expression of receptors which could be internalized or lost by shedding or affect presence of surface epitopes and thus ultimately cell surface expression of potential antigens chosen for positive selection. Therefore, if the digestion and disaggregation processes are not fully achieved, this may lead to low yields despite the high purity, which must be taken into account when performing sequencing analysis since it may influence the downstream procedure. Also, extended purification protocols aiming to reach single brain endothelial cell suspensions bear the risk of inducing changes in gene expression in the endothelial cells due to loss of the tight junction interactions, as cross-talk between mature cell–cell junctions and the nucleus are well established.

Another important factor is that the selection is based on generally accepted markers for the cell population of interest, with the above-mentioned limitations. In addition, heterogeneity in marker expression along the brain microvasculature might influence targeting efficiency. Therefore, it is good practice to refer to the most recent studies that better define different cell populations of the brain vasculature [13, 76], in order to improve the targeting strategy and the selectivity of the technique.

When using magnetic beads in a positive selection it is also important to know how, or if, to separate cells from the beads afterwards. A step that might become essential is when the isolated fraction is intended to be used in cell culture. According to the first protocols using this method of isolation, incubation of the cells in trypsin/EDTA at 37 °C releases the beads once the selection is achieved [77]. Improvements in this technique allow for the establishment of procedures which require a less aggressive approach or even do not require detachment of the beads following isolation [78], since they do not affect growth nor survival of isolated cells. Therefore, also depending on the brand, some beads have been shown to detach spontaneously from the cultured cells after several days, whereas other beads might need a DNAse treatment to break the DNA chain that attaches the microbead to the antibody [52, 70]. In any case, most of the currently used magnetic beads are completely suitable for subsequent analysis.

#### Guidelines

- Clearly indicate the antibody used to coat the beads and the rationale behind the choice; refer to recent publications (if possible) to define the cell population that will ideally be targeted.
- Clearly indicate the amount of material obtained after bead selection, including number of isolated cells and amount of RNA extracted from them; this is useful information for the study itself and for future reference.
- Clearly indicate the necessity of separating beads from cells or not, if choosing positive selection of the target cell.

### Specific separation of microvessel cell populations: fluorescence-activated cell sorting (FACS)

FACS is a powerful technique that uses flow cytometry to selectively separate cell populations from complex pools of different cells. FACS-based selection has been used in transcriptome profiling studies of the brain barriers [13, 14, 79]. Interestingly, these techniques include high purification of cells expressing fluorescent reporters in transgenic mice, however, any cell that expresses the construct will be selected, introducing possible contamination in the downstream analysis. For example, FACS has been successfully used to enrich brain endothelial cells isolated from claudin-5-GFP mice [13] or tamoxifen-treated Rosa-tdTomato; VE-Cadherin-Cre^ERT2^ mice [14]. Alternatively to fluorescent reporter mice, other studies have achieved isolation of brain endothelial cells via FACS by antibody staining prior to sorting, using fluorochrome-conjugated antibodies against CD31 [80, 81] or by a combination of antibodies against CD31 and CD13 to isolate endothelial cells and pericytes, respectively, from different microregions in the mouse brain [82].

In any case, the final sample after FACS consists of a highly enriched fraction of the cell type of interest. However, a high amount of starting material is often necessary to obtain a sufficient yield after the sorting, although it will also depend on the needs of the downstream application and/or analysis [83, 84]. Also, FACS sorting may induce an oxidative stress response in the endothelial cells that needs to be considered.

As already mentioned in other sections of this paper (e.g.: beads selection), obtaining a viable single cell suspension is also crucial when isolating cell populations of the brain barriers prior to FACS. Similar to the beads selection, a combination of mechanical and enzymatic digestion prior to FACS is often used to improve cell–cell dissociation. Indeed, a good single cell suspension reduces the amount of false positives/negatives produced by antibody staining (when not relying on reporter mice) or reporter proteins prior to FACS. In line with the necessity of a single cell suspension, duplet exclusion should be tightly controlled to ensure the best purity and reliability of the sorted material, as well as a strict gating strategy according to the experimental needs [85]. In addition, abundance of the population of interest is also critical, e.g.: if the level of endothelial cells is lower than 15% of the total, the sorted cells might not be viable. A density gradient enrichment before performing FACS could potentially solve this issue [51]. In general, FACS poses some technical challenges and a fine balance between time needed for the sorting, quality of the starting material and viability of the sorted cells needs to be experimentally tested.

#### Guidelines

- The flow conditions and the instrument used should be indicated.
- The precise scatter and fluorescence gating strategy used for the FACS of the target cell should be included in the supplement of the research article, as well as a detailed description of the isolation protocol and potential staining steps performed prior to the sorting and their duration.
- Duration of sorting itself and yield of cells received should be described.
- Time of RNA extraction following the FACS should be clearly stated, as different experimental designs implement differences in time points, e.g.: extraction of RNA right after FACS of after several hours due to travel from the sorting facility back to the laboratory.

### Laser capture microdissection (LCM)

Laser capture microdissection (LCM) allows for the dissection of CNS microvessels from a CNS tissue section with the help of a microscope and a laser. Dissected CNS microvascular endothelial cells can be later captured by adsorption, ejection, gravity or aspiration. LCM permits to take a snapshot of the transcriptomic profile of the BBB, in opposition to methodologies that require long incubation times.

One of the main limitations of LCM is the low yield of this laborious technique, which can be circumvented by using kits designed to isolate RNA from small amounts of cells [86] or by performing rounds of RNA amplification prior to downstream analysis [87]. However, using LCM to capture small cells, such as BBB endothelial cells, may be challenging, and contamination from astrocytic endfeet and/or pericytes is a major concern. In order to improve the cellular purity of the preparation, thinner sections can be used, thus decreasing the chances of including cells above or below the plane of interest. Alternatively, LCM on cross sections of vessels also provides better purity than longitudinal sections, although lower yield [88]. To aid the visualization of endothelial cells, rapid immunohistochemistry may be coupled to LCM [89], in a technique known as immuno-LCM. The reproducibility of immuno-LCM to study BBB gene expression in mice has been demonstrated [90]. This technique was further validated in human postmortem frozen [87] and Formalin-Fixed Paraffin-Embedded (FFPE) brain sections [89].

#### Guidelines

- Consider the balance between yield and purity of the isolated CNS microvascular endothelial cells when deciding to use cross sections or longitudinal sections.
- It is recommended to use RNA isolation kits specifically designed for small amounts of cells, or performing RNA amplification prior to sequencing.
- Test for and consider the possible cellular contaminants co-isolated with the BBB endothelial cells.

### BBB in vitro models: cultured primary brain endothelial cells and brain endothelial cell lines

Isolated brain microvascular fragments or single cells can be cultured and used as a BBB endothelium in vitro model. Most of the in vivo BBB characteristics are maintained by primary cultures of brain endothelial cells, hence representing powerful tools to study various aspects of BBB properties. However, often these cultures offer restricted capacity of genetic manipulation (e.g. transfection) and can be maintained in culture for a limited amount of time and/or passages. Nevertheless, numerous primary cultures of brain endothelial cells have been established from both mouse [58], rat [91] and human [92, 93] brain.

On the other hand, BBB in vitro model established by immortalized cell lines allow for much easier handling, as in many cases the cells can be cultured and passed as needed, in addition to much better tolerance to genetic manipulations. This makes cell lines a very suitable tool for high-throughput screening purposes, as they are also a much more homogenous cell population compared to primary cell cultures, where often contaminants are found in the culture. As a major drawback, cell lines of the BBB endothelium do not strictly retain BBB characteristics such as high tightness and very low permeability to the same degree as primary brain endothelial cells, therefore careful selection of the best suited BBB cell line is needed according to the specific scientific question being answered.

In both cases, the presence of additional BBB cell types found in vivo is not always modeled in the in vitro systems, such as for example the presence and anatomical disposition of the astrocytic endfeet found in the NVU in vivo. To overcome these limitations, sophisticated co- or tri-cultures of brain endothelial cells together with pericytes or astrocytes from different sources have been established and, to a certain degree, mimic the in vivo NVU structure [94–96].

In the context of transcriptomic studies, both BBB cell lines and primary brain endothelial cells cultures have been successfully used in transcriptomic approaches. For example bulk RNA-seq has been performed on the human cerebrovascular endothelial cells (hCMEC/D3) [97] and on primary mouse brain microvascular endothelial cells (pMBMECs) [79]. Interestingly, a comparative microarray analysis between freshly isolated or cultured pMBMECs with the endothelioma cell line bEnd.5 has highlighted important changes in the mRNA levels of genes associated with BBB characteristics [69].

Recent advancements in stem cell technology have furthermore allowed derivation of human in vitro models of the BBB from stem cell sources including human cord blood-derived stem cells of circulating endothelial progenitors [98] and human induced pluripotent stem cells (hiPSCs; summarized in [99]). hiPSCs derived from one individual opens the entirely novel opportunity to study BBB dysfunction from individual patients as their hiPSCs provide a scalable and renewable source for establishing brain microvascular endothelial cells. The presently available hiPSC derived in vitro BBB models are very well characterized with respect to their barrier properties and expression of BBB specific transporters and efflux pumps [100–102]. At the same time RNA-Seq analysis has shown that hiPSC derived brain microvascular endothelial cells, as most hiPSCE—derived cells, do not fully recapitulate all aspects of the BBB [98]. Present hiPSC derived in vitro BBB models, e.g. still lack expression of the full array of trafficking molecules required for immune cell interaction with the BBB.

#### Guidelines

- Consider the effect of culture-induced mRNA expression changes in the in vitro BBB models due to medium composition.
- Use RNA-Seq profiling of in vitro BBB models and especially of hiPSC- derived in vitro BBB models to benchmark them against the BBB in vivo.

### Considerations for isolating the entire choroid plexus vs choroid plexus epithelium

The ChP consists of a highly vascularized stroma populated by immune cells and is surrounded by a layer of highly specialized epithelial cells which form the BCSFB. Contrary to the BBB, the ChP endothelium is fenestrated and does not form a BBB [103].

ChP transcriptomic studies of the entire ChP tissue will include the transcriptome of the epithelial cells forming the BCSFB but also from the endothelial cells, stromal fibroblasts and immune cells of the ChP. Alternatively, the ChP epithelial cells can be isolated to focus on the transcriptome of the BCSFB.

Using the whole ChP greatly simplifies the protocol for tissue isolation, but the cellular heterogeneity within the ChP will complicate subsequent analysis and interpretation of the results especially when performing bulk RNAseq studies. However, these studies will provide additional information on the other components of the ChP, such as the vasculature or immune populations. Many RNA-seq studies have taken this approach, particularly those focused on humans [104, 105].

Alternatively, if the barrier component of the ChP is the focus of the study, the epithelial cells can be isolated. While this results in cleaner data, dissociating the ChP may be challenging (see below). To overcome these difficulties, the novel single-nucleus RNAseq method emerges as an option for tissues that are hard to dissociate, such as the ChP [42].

The research question and the technical limitations will determine whether the whole ChP or the isolated epithelium will be sequenced.

### Methods for isolating the choroid plexus epithelium

The techniques for isolating the ChP epithelial cells are similar to those for the BBB. Of note, the ChP epithelium is composed of large cuboidal cells, which are easier to dissect microscopically with LCM compared to the thin BBB endothelium. Indeed, LCM has been used to isolate the human ChP epithelium for microarray studies of the BCSFB [106–108]. In animal models, mechanical disruption of the entire ChP surgically removed from the brain ventricles is typically combined with enzymatic digestion. In order to release epithelial adherens and tight junctions as well integrin mediated adhesive contacts to the epithelial basement membrane, calcium removal is recommended, for example by using the chelator EDTA [109, 110] or calcium free medium [111], but is not an essential requirement [112]. Further purification can be achieved by FACS using an epithelial marker such as TTR [41]. However, expression of *TTR* has been recently identified in ChP macrophages [113], and the choice of markers should be done with awareness of their limitations. To our knowledge, the only human primary epithelial cells are those commercially available (ScienCell), and no isolation protocol has been published to date.

#### Guidelines

- The research question and the technical limitations will determine whether the whole ChP or the isolated epithelial cells will be sequenced. This should be specified in the methods.
- The ChP can be particularly challenging to dissociate. The techniques for isolating the ChP epithelium include LCM, mechanical and enzymatic digestion. Resulting purity should be assessed and reported.

### Pre-sequencing tissue or cell purity assessment

Before performing RNA-seq, it is good practice to ensure that the chosen isolation strategy results in the desired brain barrier cell purity. Indeed, knowledge about the degree of brain barrier cell purity is essential as it dramatically reduces possible biases in the downstream analysis, overall improving the biological meaningfulness of any RNA-seq study. Pre-sequencing purity assessments can be achieved by different techniques, often used in combination. Common contaminants when isolating endothelial cells of the BBB are pericytes or pericyte fragments and astrocytic endfeet, which can hardly be avoided (Fig. 5, Table 1). These contain RNA and are thus readily detectable by assessing expression by qPCR of specific markers such as platelet-derived growth factor receptor beta (PDGFR-β) or GFAP, respectively. Immunostaining provides information about the location of the probed proteins while flow cytometry allows for quantitative detection of the contaminants with higher sensitivity, although it requires a significantly higher number of cells compared to immunofluorescence imaging. Therefore, qPCR can be used in combination with immunofluorescence imaging in order to estimate the purity at both the RNA and protein level. All of the techniques depend, to differing degrees, on described cell markers (Table 2).

**Table 2:** Selected molecular markers of relevant CNS cell types with species and methodological information

	Endothelial cells of the BBB	Pericytes	Astrocytes	Smooth muscle cells	CP/CVO endothelial cells	CP epithelial cells	Immune cells	Oligodendrocytes	Fibroblasts
AQ1	–	–	*–*	–	–	**[**114**]** ***IHC*** [115] *IHC*	–	–	–
AQP4	–	–	[116] *IHC*	–	–	**[**114**]** ***IHC***	–	–	–
⍺-SMA	–	± IHC [117] *IHC* [118]	–	[119] *IHC*	–	–	–	–	–
CD31/PECAM1	**[**120, 121**]** ***IHC***	–	–	–	[122] *IHC*	–	**[**123**]** ***IHC***	***–***	***–***
Cdh5	[124]	–	–	–	[125] *RM*	–	–	–	–
Cldn-1	[126] *IHC*	–	–	–	–	[127*–*129] *IHC*	–	–	–
Cldn-2	–	–	–	–	–	[127, 128] *IHC*	–	–	–
Cldn-3	[130] IF [79]^a^	–	–	–	–	–	–	–	–
Cldn-5	[131] *KO + IHC/WB* **[**132**]** ***IHC***	–	–	–	–	[127] *IHC*	–	–	–
Cldn-11	–	–	–	–	–	[128] *IHC*	–	***IHC*** **[**133**]** *IHC* [134]	–
FGF									*WB* [135]
GFAP	–	–	[136] IF	–	–	–	–	–	–
GLAST	–	–	*WB* [137]	–	–	–	–	–	–
ICAM-1	[138] *IHC* **[**139**]** ***ELISA***	**[**140**]** ***ICC***	–	–	–	–	**[**141**]**	***–***	***–***
MHC-II	–	–	–	–	–	–	[142] *PCR*	–	–
NG2/CSPG4	–	[143] *IHC*	–	–	–		–	[144] *Immunogold*	–
OAP-1	***–***	–	–	–	*–*	*–*	–	[134] *WB + IHC*	–
Occludin	**[**132**]** ***IHC***	–	–	–	[127] *IHC*	[127, 128] *IHC*	–	–	–
PDGFR-β	–	[143] *IHC*	–	–	–	–	–	–	–
Podoplanin	–	–	–	–	–	[145] *IHC, WB* [146] *IHC*	–	–	–
TTR	–	–	–	–	–	[41] QPCR, RNAseq	–	–	–
VCAM-1	**[**147**]** ***ELISA***	**[**140**]** ***ICC***	–	–	–	–	–	–	–
ZO-1	[148] *IHC* [149] *ICC*	–	–	–	–	[128] *IHC*	–	–	–

Italic represents evidence in the mouse, bold italic represents evidence in humans, underline represents evidence in the mouse and humans

*IHC* immunohistochemistry, *RM* reporter mice, *IF* immunofluorescence, *PCR* polymerase-chain reaction, *QPCR* quantitative polymerase-chain reaction, *RNASeq* next generation RNA sequencing, *scRNAseq* single-cell RNA sequencing, *KO* knock-out, *WB* Western-blot, *ICC* immunocytochemical staining, *ELISA* enzyme-linked immunosorbent assay

^a^A study showing contrary evidence (absence)

Another potential source of contaminants when isolating endothelial cells of the BBB are ChP cells, in particular the ChP epithelium. Indeed, in the vast majority of the preparation the ChP is not removed from the processed material, therefore potentially accounting for contamination. Performing qPCR for choroid plexus specific markers such as transthyretin or keratin-8 will allow to determine the presence of ChP mRNA in the brain endothelial preparation. For example, low expression of claudin-3 mRNA has been reported in freshly isolated brain microvessels, despite recent evidence proving lack of claudin-3 expression in the mouse brain microvasculature. This could be due to contamination of the isolated brain microvessels with ChP epithelial cells, which express claudin-3 [79].

### Isolation and purification of RNA from BBB endothelial cells or microvessels

**Fig. 6:**
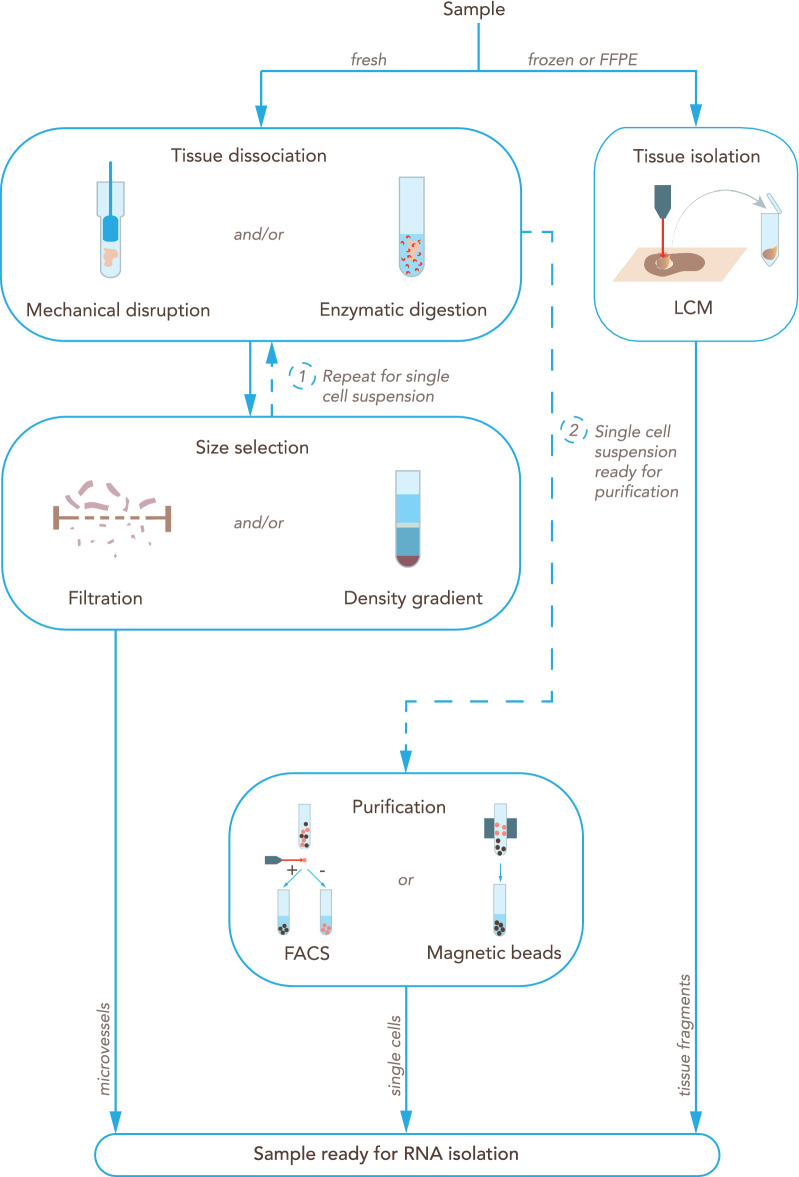
Overview of the main steps for processing a CNS tissue sample into BBB-related material ready for RNA isolation. Fresh samples are dissociated by mechanical disruption, enzymatic digestion, or a combination of both. Typically, tissue is first mechanically disaggregated into smaller pieces to facilitate the exposure to the enzyme solution. Dissociated tissue is then selected according to size by passing through one or a series of filters, by a density gradient, or both. This process isolates the microvessels. For isolating single barrier cells, tissue dissociation (particularly enzymatic digestion) can be repeated [1] after the initial size selection steps. The single cell suspension can be further purified or enriched for certain cell types [2] by using a fluorescence-activated cell sorter (FACS) or magnetic microbeads labeled with an antibody against a cell marker. Alternatively, if the tissue of interest is frozen or formalin-fixed paraffin-embedded (FFPE), a common approach is to isolate the microvessels by laser capture microdissection (LCM)

RNA isolation methods have to be chosen depending on the type and availability of starting material, on the one hand, and the intended RNA-seq analyses, on the other (Fig. 6). The use of inappropriate RNA isolation methods can result in low quantity and/or quality of RNA and consequently in less accurate and irreproducible results or even in complete failure of the analysis [150].

Contaminations with extrinsic RNA and DNA, or with nucleases that might lead to the degradation of RNA samples can have a negative impact on the results. General measures to avoid these issues include thorough and regular cleaning of work areas and equipment with decontamination solution as well as use of clean gloves, aerosol barrier pipette tips, and DNAse and RNAse-free plasticware. Additionally, it is recommended to carefully handle RNA samples at the temperature suggested by the manufacturer of the isolation kit [151].

Commercially available RNA isolation protocols generally follow two main steps: (i) sample lysis, homogenization and clearing, and (ii) RNA purification. Isolation kits and protocols must be chosen according to the type of sample (e.g. cell culture, frozen tissue, FFPE tissue, etc.) and RNA molecules to be purified (e.g. small or large RNA molecules).

### Sample lysis, homogenization and clearing

**Fig. 7:**
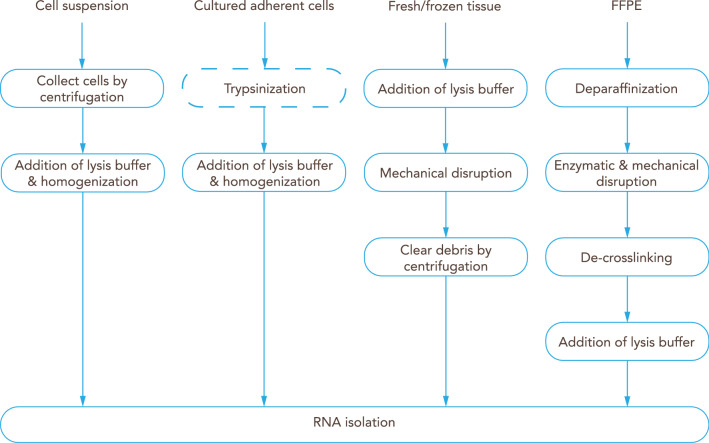
Overview of commonly used RNA isolation protocols. Preparation of BBB-derived samples according to the type of sample. Cells in suspension are first collected by centrifugation, while adherent cultured cells are commonly trypsinized; then lysis buffer is added and cells are homogenized before proceeding to isolation of the RNA. Fresh frozen tissue can be mechanically disrupted in lysis buffer; debris should be removed by centrifugation before RNA isolation. Formalin-fixed paraffin-embedded (FFPE) tissue is first deparaffinized, and tissue disruption can be achieved by enzymatic (proteinase K) and/or mechanical means; de-crosslinking is followed by addition of lysis buffer, and then RNA is isolated

Cell lysis is commonly performed using a guanidine-thiocyanate-based buffer combined with a strong reducing agent, such as tris(2-carboxyethyl)phosphine hydrochloride, 2-mercaptoethanol or dithiothreitol to ensure the complete cell lysis and protein denaturation (lysis buffer), but these procedures vary according to the characteristics of the starting sample (e.g. adherent cell culture, cell suspension, frozen tissue or formalin-fixed paraffin-embedded (FFPE) tissue)—(Fig. 7).

#### Cultured adherent cells

Cultured adherent cells, like endothelial cells, can be subjected to trypsinization prior to cell lysis or they can be lysed directly in the culture container by replacing the liquid medium with lysis buffer directly to the cell monolayer. Cell lysis by addition of lysis buffer is recommended to be performed prior to storage to avoid transcriptomic alterations during freezing. The cell lysate is safe to be stored at − 80 °C.

#### Cell suspensions

Cell suspensions, such as microvessel fragments or single endothelial cells, can be pelleted by gentle centrifugation (≤ 500×*g*). After complete removal of the supernatant, the cell pellet is re-suspended in lysis buffer. As for the lysis of adherent cells, cell lysis is recommended to be performed prior to storage. Sorted and isolated single cells can be directly collected in lysis buffer and mild lysis buffer, respectively.

#### Tissue samples

At the moment of collection, tissue samples, like the ChP, are recommended to be stored in a RNA stabilization solution or to be snap-frozen in liquid nitrogen. Samples in stabilization solution can be stored up to 4 weeks at 4 °C or at − 20 °C for long-term storage [152]. Samples snap-frozen in liquid nitrogen are safe to be stored at − 80 °C for more than 20 years [153]. Tissue samples might need to be disrupted using different techniques, such as the TissueRuptor, TissueLyser, ZR BashingBead Lysis Tubes or thorough grinding under liquid nitrogen using a mortar and pestle [152]. Remaining tissue and other precipitates might need to be removed by centrifugation and the supernatant can be used for subsequent RNA isolation.

#### FFPE tissue samples

FFPE tissue samples derived from brain microvessels must be subjected to deparaffinization using xylene or other commercially available solutions. Subsequently, tissue and protein digestion is performed using proteinase K. Next, formaldehyde-derived crosslinks of nucleic acids and proteins must be reversed by incubating at more than 80 °C. Finally, the sample might be cleared by centrifugation and the supernatant can be used for subsequent RNA isolation [154].

### RNA purification

During experimental design and before performing RNA purification, it is necessary to identify which RNA molecules are relevant for the research question. Messenger RNA (mRNA) is the RNA that will be translated by the ribosomes into proteins. mRNA is characterized by having a coding sequence surrounded by 3′ and 5′ untranslated regions and a long sequence of adenine nucleotides at the 3′ end (poly-A tail). Several other types of non-coding RNA have important roles in cell biology, e.g. ribosomal RNA (rRNA) and transfer RNA (tRNA) are necessary for the translation process. Additionally, other RNA families are important for gene expression regulation as for example, microRNA (miRNA) with a size of ca. 22 nucleotides, other small RNAs (< 200 nt) and long non-coding RNA (lncRNA) with sizes greater than 200 nucleotides. Regarding the RNA content in a cell, it is important to notice that just mRNA and many lncRNA have a poly-A tail at the 3′ end. Additionally, rRNA represents the majority of the RNA content in the cell.

The combination of a highly concentrated chaotropic salt (e.g. guanidinium thiocyanate) in the lysis buffer with a certain concentration of an organic solvent (typically ethanol or isopropanol) allows the adsorption of nucleic acids to the silica matrix in spin columns. Although this solid phase extraction allows for efficient and easy isolation of purified nucleic acids, several details must be taken into consideration. The final concentration of ethanol or isopropanol in the mixture with lysis buffer is essential to promote the adsorption of RNA to the silica matrix [150]. Different ethanol or isopropanol concentrations result in the isolation of RNA molecules with different sizes, e.g. small RNA molecules (containing miRNAs) with a size between 16 and 200 nucleotides and large RNA molecules (containing mRNA and lncRNA) with a size greater than 200 nucleotides. Therefore, it is necessary to carefully select the applied protocols and correspondent ethanol/isopropanol concentrations before starting the isolation. Due to the possible impact of genomic DNA (gDNA) contamination in RNA-seq analyses, thorough digestion of gDNA remnants in RNA samples is mandatory. gDNA removal columns or integrated on-column DNA digestions are included in most RNA isolation kits [152].

#### Guidelines

- The RNA extraction protocol should be selected based on tissue type and quantity, as well as the intended sequencing and analysis.
- Specific protocols are required for the isolation of total RNA including miRNA.
- Correct sample homogenization and clearing are essential for isolation efficiency of RNA and analysis reproducibility.
- Genomic DNA contamination can have a considerable impact on the sequencing results therefore, gDNA removal or digestion is mandatory.

### RNA quantification and quality control

RNA concentrations are best determined using a fluorometric quantification or qPCR. For example, fluorometric quantification can reliably measure RNA concentrations as low as 0.2 ng/μl. Spectrophotometric quantification is not recommended due to its inaccuracy, especially for small amounts of RNA, and due to the unreliable results in the presence of contaminants with an absorption wavelength close to those of DNA and RNA, e.g. phenol. Nonetheless, the spectrophotometer can be a useful tool to determine contaminations. For example, measurements with absorption wavelengths of 230 and 280 nm can indicate contaminations with guanidinium salts and proteins, respectively.

Quality control is best performed using an automated capillary electrophoresis platform that calculates a score for the RNA quality. Depending on the automated capillary electrophoresis platform used for RNA characterization, the RNA quality score might have different names (e.g., RNA integrity number, RNA Quality Score, RNA Quality Number, etc.). The RNA quality score has values between 0 (poor quality) and 10 (good quality) and it is calculated using an algorithm that incorporates several features of the RNA electropherogram, such as the ratio of 28S:18S ribosomal RNA [155]. A quality score for total RNA higher than 8 is recommended for the majority of RNA-seq library preparation techniques. In case of FFPE tissue, samples with score values around 2 can be used to perform RNA-seq using specific protocols for library preparation [156, 157]. RNA quality scores of the respective RNA isolates are often missing in RNA-seq datasets in public databases, such as Gene Expression Omnibus (GEO—https://www.ncbi.nlm.nih.gov/geo/) or Sequence Read Archive (SRA—https://www.ncbi.nlm.nih.gov/sra). Given their impact in study reproducibility and cross-study meta-analyses it is advisable to include these in publications as well as public databases.

Human BBB and BCSFB samples from a clinical setting often present suboptimal preservation, which may affect the resemblance of the transcriptome to the in vivo situation. With some exceptions [158–163], the use of biopsies from human brains for RNA-seq is uncommon and postmortem material is used instead. Two main factors should be considered when using postmortem brain or spinal cord samples, namely the premortem agonal state of the patient and the postmortem delay until sample collection. Prior to death, the patient may have suffered from fever, sepsis or hypoxic changes, as well as the provision of oxygen, which will strongly and selectively affect the levels of certain mRNAs [164]. Postmortem delay of tissue retrieval and preparation hampers RNA integrity as a result of transcript degradation, possibly in a non-random way [165]. RNA integrity can strongly affect transcript levels [165]. Particularly, low RNA integrity samples present an upregulation in translation‐related pathways [166]. The time to sample preservation should be minimized, but this is usually not in the hands of the researchers. Samples with low RNA quality can be excluded by using a certain threshold (as measured by the RNA Integrity Number or RIN), or a mathematical model can be applied that accounts for the differential decay of different transcripts, thus increasing the statistical power [165].

Other factors that may lead to RNA degradation are the handling and storage conditions and, if applicable, the sectioning process. However, there are reports of a remarkable RNA stability in postmortem human brain samples [167, 168].

In summary we advise the starting material to fulfill three criteria:

- The RNA quality should be accessed, and the library preparation protocol should be chosen accordingly.
- The quantity of isolated RNA must be sufficient to perform library preparation. Commercially available kits allow for preparation of libraries with 0.1 to 500 ng of total RNA.
- The RNA samples must be clear of contaminants such as proteins, salts, sugars or DNA.

#### Guidelines

- Integrity values for RNA are essential both for the selection of the enrichment method as well as sample selection.
- Assess RNA quantity and quality of the samples prior to sequencing. A quality threshold should be debated between the wet-lab researcher, the sequencing technician and the bioinformatician.
- If the data is made publicly available, always disclose the integrity values of the included samples.
- Consider the integrity of the samples when choosing the library preparation method (discussed below).
- Consider pre- and postmortem factors: they may affect heterogeneity in the whole transcriptome.

### Design and preparation of sequencing libraries

When designing an RNA-Seq experiment, the following aspects must be considered: sequencing strategy, library preparation, number of replicates and sequencing depth (Fig. 3).

The sequencing strategy is important to guarantee the quality of the analysis at a reasonable cost. Paired-end sequencing means sequencing from both ends of the cDNA fragment. Since both fragments are aligned as a pair of reads, this strategy is preferable for de novo transcriptomic assembly, to study isoform expression or poorly annotated transcriptomes. Single-end sequencing implies that just one end of the cDNA fragments is sequenced. This approach is more cost-effective than paired-end sequencing and it is suitable for studying gene expression in well annotated organisms, such as human, mouse, rat and zebrafish. Additionally, the length of the reads produced during sequencing can be chosen depending on the purpose of the study. Longer reads (from 150 to 300 bases) are more cost-intensive but offer a higher resolution to study alternatively spliced RNA isoforms or poorly annotated transcriptomes. Shorter reads (e.g. 75 base pairs) are suitable to study gene expression in well annotated organisms.

The sequencing depth (number of sequenced reads per sample) used in RNA sequencing experiments is important for detection of differentially expressed genes, especially for lowly abundant transcripts. RNA-Seq experiments are recommended to use a minimum sequencing depth between 10 and 30 million reads per sample [169, 170]. However, in human samples, it was shown that increasing sequencing depth above the ten million reads per sample threshold does not improve the identification of differentially expressed genes as much as increasing the number of replicates [171].

Inclusion of replicates in RNA-seq experiments is important to assess technical and biological variations:

- Technical variability in sequencing is usually low. Nonetheless, the technical variability introduced during sample collection and library preparation can be estimated using technical replicates or RNA spike-in like the one developed by the external RNA controls consortium (ERCC) [172] The ERCC RNA spike-in is a mixture of 92 synthetic RNA molecules and each molecule has a defined concentration. This mixture can be used to technically validate sequencing results and estimate technical variability.
- Biological variability is the natural variation due to physiologic differences among subjects or over time in the same subject. Biological variance is usually more pronounced than technical variance and must be addressed with greater concern. Biological variability can be observed at different levels according to the sample type (e.g. cell line, mouse strain or human tissue). For instance, while studying the BBB from human samples, biological heterogeneity might be introduced by several variants, such as genetic background, lifestyle, hormonal level, medical history, sex or age. Differently, when studying the BBB from animal models (e.g. mouse), the variants that might introduce biological variability are mostly age, sex and strain. This reduced number of variants is expected to introduce less biological variability in samples from animal models when compared to samples from humans. Although the variability introduced from such variants can be minimized by using, inter alia, sex and age matched samples, uncontrollable biological heterogeneity should be accounted for by using biological replicates. For example, human BBB samples cannot always be collected in the same stage of the male or female hormonal cycles. Since it is known that hormones like steroids and estradiol regulate BBB permeability and tight junction protein expression, respectively, it is important that such biological variability is taken into consideration by using biological replicates [173]. Against this backdrop, the ideal number of biological replicates must be increased when studying very heterogeneous samples, species or strains. Currently, most RNA-Seq experiments include at least three biological replicates. A recent study using human whole-blood RNA-Seq data shows that the power to detect differentially expressed genes (twofold or higher change) is 87% and 98% using three or five biological replicates, respectively. Using three or five replicates, the ability to detect smaller changes in expression (1.25-fold) is 17% and 25%, respectively [174]. Therefore, a sequencing depth of 10 million reads and a minimum of 3 to 5 replicates are recommended to reliably detect major changes in gene expression. Since adding more replicates is more beneficial than increasing sequencing depth, the use of 12 replicates is recommended to detect minor changes in gene expression (e.g. 1.25-fold) [170, 175, 176].

Since ribosomal RNA makes up most of the cellular RNA, rRNA depletion or mRNA enrichment should be performed prior to library preparation. Ribosomal RNA depletion or mRNA enrichment methods must be carefully chosen depending on the RNAs of interest and the integrity of the RNAs. For instance, ribosomal RNA depletion can be preferable for degraded material if poly-A bias is observed.

**Fig. 8:**
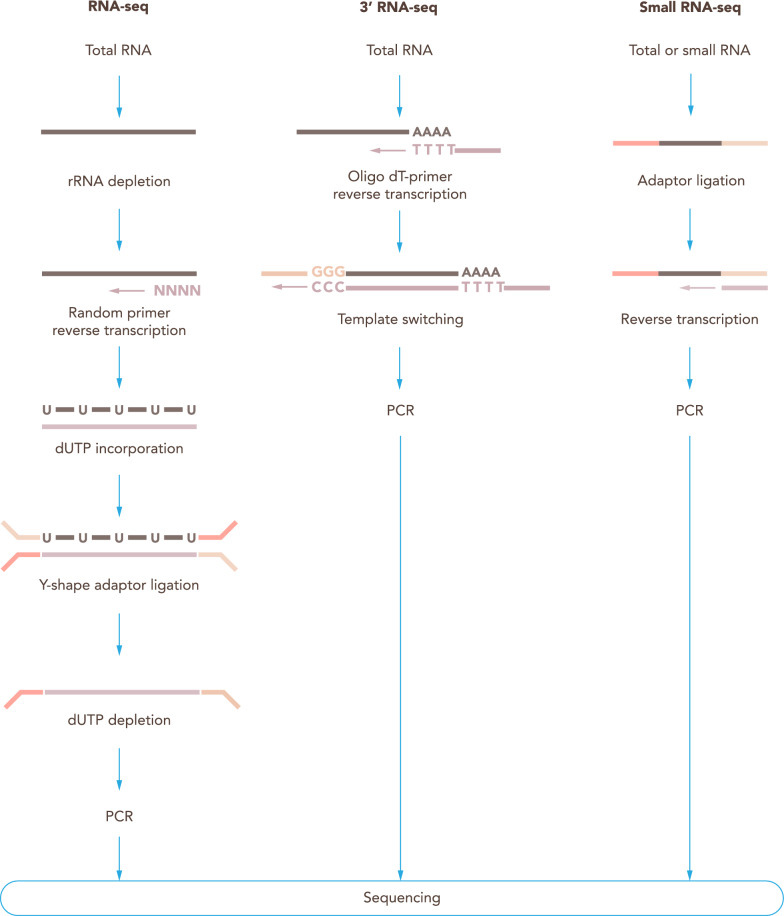
Library preparation protocols. Commonly used library preparation protocols for RNA sequencing. For RNA-Seq, a first step of ribosomal RNA (rRNA) depletion from the total RNA is performed; random primers are used for reverse transcription; dUTPs are used for the second strand synthesis; Y-shaped adaptors are then ligated, and then the second strand containing dUTPs is depleted, allowing to retrieve stranded information. 3′ RNA-Seq makes use of oligo dT primers for the reverse transcription, which selects mRNA. Using template switching, the second adapter is incorporated in the cDNA molecules. Small RNA-Seq uses adaptors that ligate to the small RNAs and allow the reverse transcription

It is important to understand and choose carefully between stranded and unstranded library preparation protocols. Stranded RNA-Seq library preparation is characterized by the use of deoxyuridine triphosphate (dUTP) instead of deoxythymidine triphosphate (dTTP) during the second strand synthesis. Thanks to this alteration, the second strand can be degraded using uracil-N-glycosylase prior to the PCR amplification and only the first strand of cDNA will be amplified and sequenced (Fig. 8). A stranded RNA-Seq library retains the information of the template DNA strand from which an RNA was synthesized. Stranded RNA-Seq performs better in handling read ambiguity in overlapping genes transcribed from opposite strands and identifying antisense transcripts. Therefore, strand-specific RNA-Seq is preferred to unstranded protocols. However, when well-annotated genomes are available (e.g. human or mouse) or when analyzing samples with low RNA input, unstranded RNA sequencing can be considered nonetheless [177]).

Although not the main focus of this manuscript, since they have important applications in the field of brain barriers, we also want to briefly highlight 3′ RNA sequencing and small RNA sequencing protocols.

3′ end RNA sequencing is an alternative to RNA-Seq. Library preparation techniques are almost identical but unlike RNA-Seq, 3′ end RNA sequencing includes an enrichment step that, following fragmentation, excludes all but the outermost 3′ fragments adjacent to the poly-A tail. Due to the information lost during the enrichment for terminal fragments, 3′ end RNA-seq is not suited for studying open reading frames and, consequently, alternatively spliced transcript isoforms. Since 3′ end RNA-Seq generates exactly one read for each transcript and allows the incorporation of unique molecular identifiers (UMI) as applied in the Massive Analysis of cDNA Ends (MACE) method [178]. The technique also quantifies alternative poly-adenylation events which are important for mRNA properties such as stability [179]. Notably, 3′ end RNA sequencing generates reliable transcriptome profiles also from poor-quality samples and is currently the main method used in single cell RNA-Seq [180]. Thus, this technique can be recommended for quantification of gene expression and consequently for determining the downstream molecular and cellular mechanisms essential for brain barriers differentiation and function [181].

Several studies have shown the important role of small RNA in the molecular mechanisms that control the function of the BBB [182]. For example, the microRNA miR-27a-3p was identified in intracerebral hemorrhage patients to regulate BBB function and edema formation via up-regulation of AQP11 [183]. Therefore, small RNA sequencing is an important tool to better understand the role of small RNAs in the BBB. However, sequencing of smaller RNA molecules (e.g. microRNA, piwi-interacting RNA, etc.) cannot be performed using regular RNA-Seq library preparation techniques because of their relatively small size. Small RNA-Seq library preparation is usually performed by ligation of adapters to the RNA molecules followed by reverse transcription. The most recent kits for library preparation avoid generation of adapter-adapter by-products and allow the use of minute amounts of starting material.

#### Guidelines

- Correct RNA quantification and quality control are essential to ensure the quality of the sequencing results and their reproducibility.
- Strandedness and sequencing strategy have a big impact on the analysis and the results. For that reason, they must be carefully chosen.
- Biological replicates are essential and minimum sample size is affected by extrinsic and intrinsic factors.
- The selection of the correct library preparation technique is crucial for accurate analysis and should be carefully chosen according to the topic under research.

### Sequencing platforms

After completion of the first human genome sequence, the demand for cheaper and faster sequencing methods accelerated the development of NGS. Nowadays, NGS platforms enable low-cost and high-throughput analyses by sequencing billions of reads in parallel. Two of the currently most used short read sequencing methods in research labs are Ion Torrent (LifeTechnologies) and Illumina sequencing [184]. Although both technologies rely on sequencing-by-synthesis, Illumina platforms detect the fluorescence generated by the incorporation of fluorescently labeled nucleotides during DNA synthesis, while Ion Torrent platforms detect pH changes induced by the release of a hydrogen ion during the incorporation of a nucleotide into a growing strand of DNA [185, 186]. The advantages of Ion Torrent platforms are the less expensive equipment and the relatively short run time (as low as 2 h per run), while its disadvantages are increased error rates while sequencing homopolymers, lower throughput (up to 80 million reads) and a higher cost per base. In contrast, Illumina platforms require expensive equipment and a running time up to 3.5 days. The advantages of Illumina platforms are the high-throughput capabilities (up to 20 billion reads per run) and the relatively low cost per sequenced base [184]. While Ion Torrent platforms can just perform single-end sequencing with read sizes around 200 and 400 base pairs, Illumina sequencing platforms have options to perform single-end or paired-end sequencing and to sequence reads between 50 and 600 base pairs. Since Illumina and Ion Torrent have similar capabilities to detect differential gene expression between samples [187], the choice of the best suited platform should be based on the sequencing strategy, cost and time. Additionally, several other technologies can be used according to the research question at hand. For instance, single-molecule real-time sequencing technology allows the sequencing of reads with lengths up to 100,000 bases and therefore offers a valuable tool to study alternative splicing events [188]. The high cost of this technology and the high cost to perform expression profiling studies are its major limitations. Nanopore sequencing technology offers an alternative to sequence long reads. This technology uses a small and portable sequencer and can sequence reads with length up to more than 2 million bases [189]. Although the sequencing throughput is very low, the cost of the sequencing devices and reagents is also relatively low.

### Guidelines

- Full transcript RNA sequencing can be performed using nanopore or single-molecule real-time sequencing and might reveal the impact of alternative splicing variants in the BBB or BCSFB function.
- Gene expression profiling studies from BBB or BCSFB samples can be performed using both Illumina and Ion Torrent platforms.
- Sequencing technology and platform should be chosen according to four main criteria:
  - Sequencing strategy (single-end or paired-end).
  - Read length (e.g. 50, 75, 150, 300 base pairs).
  - Desired thought-put.
  - Run time..

### Data Analysis: Where to focus

**Fig. 9:**
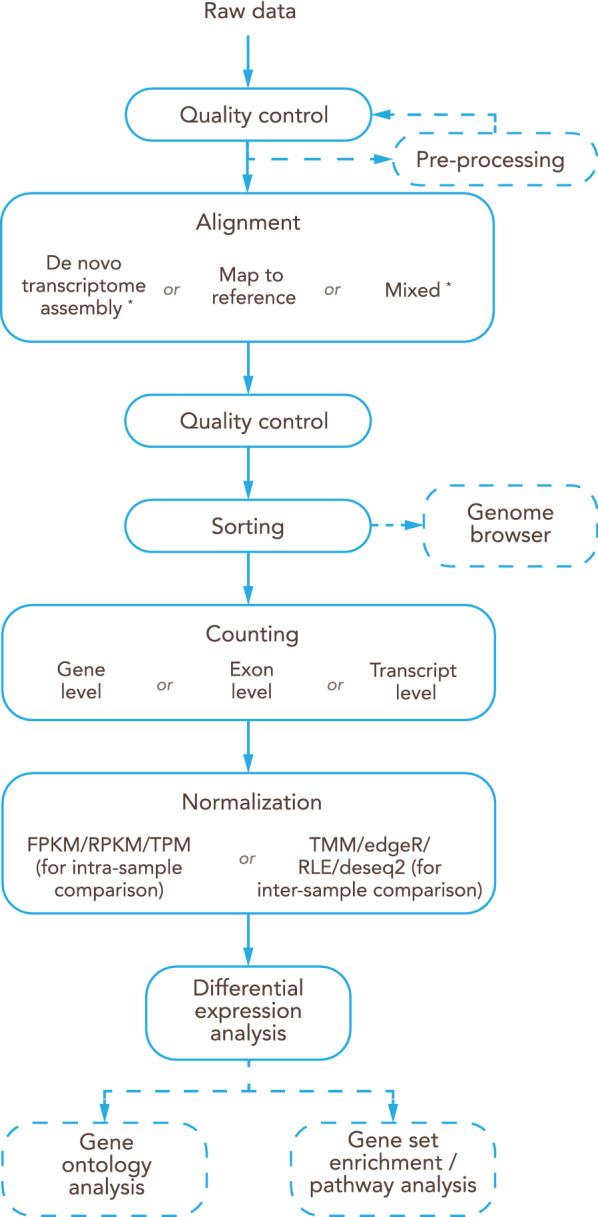
Overview of the main steps for RNA-Seq data analysis. Raw data goes through quality control steps and, if necessary, pre-processing steps are implemented. Next step is the alignment, most commonly in brain barriers studies being through mapping to reference. One more round of quality control is recommended based on the metrics of the alignment. After sorting, the files can be indexed and visualized in a genome browser. Counting can be attributed at different levels (gene, exon or transcript) and there are multiple algorithms for normalization, both in cases of inter-sample and intra-sample normalization. Finally, after the differential expression analysis, further information can be obtained with steps like gene ontology analysis, gene set enrichment analysis or pathway analysis

Data analysis for RNA-Seq is a multi-step process that can be achieved with a plethora of technologies and strategies. A proper analysis should take into consideration all the steps mentioned throughout this manuscript as they can influence the results and their interpretation. There is no optimal “one size fits all” pipeline to be used for all different transcriptomic projects in the field of brain barriers, although the overarching steps will mostly be the same (Fig. 9). Analysis are often divided into Upstream and Downstream. For the purposes of the current manuscript, Upstream Analysis consists of the raw FASTQ files to the Count Matrix Table and Downstream Analysis comprises everything after that.

A major key for a successful data analysis is clear communication between the wet lab brain barriers researcher, the sequencing facility staff and the bioinformaticians allowing for appropriate considerations of the respective limitations for optimal achievement of the project goals. We believe that there are several good publications and resources on how to optimize an analytical pipeline and benchmarking the different tools [174, 190]. As such, we will not discuss it in utmost detail, but we will instead focus on some of the steps that we have experienced to be often overlooked and we will provide some recommendations.

### Quality control and data pre-processing: often neglected, always important

For the purposes of this paper we will start after the demultiplexing process with the FASTQ files. These are the raw files that sequencing facilities and companies most commonly give to the researchers.

Along with the FASTQ files, researchers usually also get a text file with hashes (string of unique characters, in this case) for each of the FASTQs. These can be created with different algorithms (i.e. md5sum, checksum) and are used to ensure that the files were not corrupted during transfer. As such, these hashes should always be used to check the integrity of the FASTQ files.

Some companies also do some pre-processing steps, for instance trimming reads with low quality (in Phred64 or Phred33 of the Phred log scale, depending on the version and type of the sequencing platform). It is important to check if these steps were implemented, and if the answer is no, which steps should be. Removing low quality reads, for instance, can improve the efficiency of the analysis while also removing possible errors.

The most commonly used software for the effect of pre-alignment quality control (QC) is FastQC [191]. It is easy to use (by command line or graphical interface) and creates intuitive reports (in PDF and HTML format). These reports contain information about quality score encoding, the number of reads per sample, reads size, presence of contaminants, adapter sequence as well as low quality reads and the quality of the reads per position. A drop in quality is common both at the beginning and mostly at the end of the read and in paired reads; Read 2 (paired reads consist of Read1 and Read2) will generally have lower quality.

Based on this information it should be decided if the raw data is ready for alignment, if it requires pre-processing, such as removal of repetitive sequences, or if the sequencing or pre-sequencing steps need to be optimized and/or repeated.

After mapping the raw data (see next section), a genome browser, such as Integrated Genomics Viewer (IGV) [192], can be used to visualize the aligned reads. Tools like IGV [193] allow for a graphical visualization of the BAM files. Importantly, the same version of the reference genome as the one used to map the raw data must be selected. Although not its primary purpose, tools like this provide an optic way of visualizing issues such as a poly-A bias. A poly-A bias (also known as 3′ Bias) is most common in cases of RNA degradation, which is expected with the use of human post-mortem samples from the CNS, as previously mentioned.

Mapping will also generate metrics regarding overall alignment rates, reads aligned to genes, reads aligned uniquely versus reads aligned multiple times and unmapped reads. A close look at these metrics can uncover problems such as sample contamination or DNA still present in the sample.

Finally, for post-differential expression analysis, one can perform a batch effect test and, if necessary, a batch effect correction based either on known variables (samples sequenced or prepared on different days, for instance) or blindly. Plot visualization is also very informative regarding sample variability, by means of PCA or heatmaps colored by group and identified by sample, as an example.

#### Guidelines

- Quality control must be performed at every step in the analysis process.
- There are no hard rules on quality control metrics, though all applied quality measures must be mentioned in the publication of the data.
- Mapping metrics can inform about problems not detected at the raw data level and therefore should be taken into account when analyzing the data.
- Data visualization (either post-mapping or post-differential expression) can be informative regarding read distribution and sample variability, respectively.

### Overall steps: how to get the desired information from your transcriptomics study

While analytical design can be modified to serve the purposes of the analysis, most RNA-Seq experiments with the objective of identifying differentially expressed genes will follow the same general workflow.

From the raw files, the data is aligned, either by means of mapping to an available and curated reference genome or by means of performing a de novo transcriptome assembly. In the field of brain barriers, the vast majority of experiments are done using vertebrate, well established animal models, human tissue or in vitro models of the BBB and BCSFB, including cell line models. As such, usually, the alignment is done by means of mapping to a reference genome of the species of interest. There are multiple sources for reference genomes (NCBI—https://www.ncbi.nlm.nih.gov/refseq/ and Ensembl—https://www.ensembl.org/index.html—being the most used for the referenced species) as well as multiple versions and builds. When writing a manuscript, it is imperative to reference the source, version and build used for the reference genome and annotation or precise replication will not be possible.

The next major step is quantification, where the mapped reads are counted by coordinates and then grouped. This grouping can be done at either gene level, exon level or transcript level, depending on the objective of the study (i.e. is alternative splicing of interest?). This choice should be mentioned in publications in order to allow the possibility of replication.

After quantification, the count matrix contains the raw counts, which need to be normalized. Several normalization algorithms are commonly used with different purposes and efficiencies. FPKM (Fragments Per Kilobase of transcript per Million mapped reads) and RPKM (Reads Per Kilobase of transcript per Million mapped reads) are commonly used for intra-sample comparison by normalizing for both gene length and library depth. However, they have been shown to be inconsistent when compared to other methods such as TPM (Transcripts per Million), and as such, if intra-sample comparison is the objective, the latter has proven more robust [194]. On the other side, if inter-sample comparisons are the objective, the two most commonly used methods are TMM (Trimmed Mean of M-values) from edgeR [195] and RLE (Relative Log Expression) from DESeq2 [196]. Both of them forego the gene length normalization aspect as it is irrelevant (inter-sample comparison compares the same gene across samples, so they will have the same gene length). TMM and RLE have been shown to be consistent and to have good performance [197, 198]. However, there are differences between them, even if the overall concepts are similar. Recommendations for use are based on sample size, with cases of more than 12 biological replicates, DESeq 2 is recommended, while for 12 or fewer replicates, both can be used [176].

With the normalized values calculated, the following steps are the differential expression analysis and the gene ontology (GO) and pathway analysis. We will discuss cut off values and their importance in the next section and briefly mention GO and pathways.

#### Guidelines

- Always reference the source, version and build of the reference genome and annotation.
- Indicate the counted feature (Exon, gene or transcript).
- Normalization of the counts is mandatory. The selected normalization algorithm needs to take into account the different strengths and utilities.

### Differential expression analysis: cut-offs, candidate selection and cell/tissue purity assessment

After the differential expression analysis to select differentially expressed genes (DEG) a common approach is to apply cut-offs to the statistical values obtained, usually a measure of probability of a false positive, one of relative expression and one of absolute expression. One of the statistical values obtained is a p-value. These values should not be used for DEG selection. P-values are obtained for comparison between groups on a per gene basis without taking into account the number of genes. However, when undergoing parallel multiple comparisons (as is the case with tens of thousands of genes) the likelihood of obtaining false positives will increase. To appreciate this, it is necessary to understand what p-values actually represent. A p-value is merely a statistical probability that the result/observation for that gene would be at least as extreme (the observed difference would at least be the same) if the null hypothesis was true (that there is actually no difference between the two conditions for that gene). As such, if we have a p-value of 0.01 but 20,000 genes tested, it would be expected that 200 of those genes were false positives.

In order to solve this issue, a multiple testing correction is applied. There are a variety of algorithms, some of them very strict. Bon Ferroni is probably the simplest and the strictest one. It consists of dividing the α (chosen significance level, usually 0.05 or 0.01) by the number of tests (genes) to get a new α. However, while increasing confidence on the genes that do pass the new significance threshold (increased True Positive Rate) it is probable that there is a concomitant increase of the false negative ratio. A more appropriate correction is the Benjamini–Hochberg correction [199], which produces an adjusted p-value that can then be judged on the same initial α, most predominantly known as false discovery rate (FDR).

Another option is to ignore the p-value (adjusted or not) and focus solely on the fold change. Massively simplifying it, the fold change indicates the order of magnitude of the difference for that gene between conditions. This allows for the discard of genes where there are small differences that may be difficult or impossible to validate by qPCR and especially at the protein level, due to problems pertaining to low depth and the RNA–Protein abundance discrepancy [200]. Also, it should be mentioned that thresholds for log2FC (the base 2 logarithmic transformation of Fold Change that allows one to discern the direction—up and down—of regulation) are not as well defined and accepted.

A commonly used solution is to apply cut-offs for both the FDR and the log2FC, potentially also combined with an extra cut-off for overall expression.

If the goal of the experiment is mechanism or process discovery, pathway and enrichment analysis can provide guides of where to proceed in the functional validation. However, there are a few issues that we found problematic and should be considered.

Firstly, these analyses are heavily dependent on the quality of the respective databases used. For instance, the more a respective field is studied (e.g. cancer) the more information about that field is included in the database. The corollary is that the less that is known in a specific research field or about a molecule or mechanism, the less information will be available [201].

Secondly, there is a trend to use commercial tools for pathway and gene ontology analysis. While the tools appear to be solid, their restricted availability complicates reproducibility of the results. Thus, we recommend the use of open source tools for the same purpose instead.

Finally, while it is possible to have an estimation of cell and tissue purity based on the results from transcriptomics studies, the methodologies to do just that should not be relied upon. The advent of extensive datasets, such as the one from Vanlandewijck and colleagues [13] can provide useful and necessary data points but will need to be supplemented with deep bulk RNA-Seq datasets. This is especially true for the expression of genes such as claudin-3 and claudin-12, which were previously thought to be signature claudins of the BBB, which has recently been disproven or put in another perspective [79, 202].

#### Guidelines

- In RNA-Seq studies p-values require multiple test correction. However, overly harsh multiple test correction can increase the False Negative Ratio of differentially expressed genes.
- The use of individual cut-offs and or mixes have their strengths and should be used in accordance to the objective.
- The intrinsic limitation of pathway and enrichment analysis should be taken into account.

### Validation of transcriptomics results: Challenges and recommendations

It is often requested by journals or reviewers to validate RNA-Seq results in two ways. The first one is by qPCR. This RNA level validation can be performed either on the same samples as the RNA-Seq or on different ones. It is an open debate if qPCR validation is actually necessary. It is our opinion that, while not absolutely necessary, qPCR validation can provide valuable information. When done on the same samples, qPCR can validate the sequencing process, while validation of the results using a different set of biological replicates can help confirm the results [203].

The other validation approach is at the protein level. This can be achieved by different methodologies including Western Blots (WB), immunohistochemistry (IHC) and immunofluorescence (IF) staining. WBs tend to be more informative when it comes to quantification values, IHC and IF staining on the other hand allow for cellular and subcellular location of the protein being investigated. Protein level validation should be performed prior to functional studies based on the chosen candidates from the RNA-Seq results. Common issues are the absence of correlation between protein and RNA levels or of the differential expression detected amongst groups due to the RNA–protein discrepancy [200].

### Data storage and availability

Upon publication of the manuscript, all relevant data should be made accessible. This necessarily includes the raw data but can also include processed data and any relevant metadata. There are a number of specialized data repositories, including GEO (https://www.ncbi.nlm.nih.gov/geo/), SRA (https://www.ncbi.nlm.nih.gov/sra) and the European Nucleotide Archive (ENA—https://www.ebi.ac.uk/ena). As most of the data repositories allow for the data to remain private until publication, it is recommended that the process of uploading the data is done in advance. This also allows to provide the reviewers with access tokens. Outside of public data repositories, local copies of the data should also be stored and maintained.

As a complement, some groups have in addition created web-interfaces allowing other researchers to explore their results. This includes BBBomics (http://bioinformaticstools.mayo.edu/bbbomics/) [97], the Vascular Single Cells Database (http://betsholtzlab.org/VascularSingleCells/database.html) [13, 76], Brain RNA-Seq (https://www.brainrnaseq.org/) [204, 205], Single Cell Analysis of Mouse Cortex (http://linnarssonlab.org/cortex/) [206] and the Allen Brain Map (https://portal.brain-map.org/). These web interfaces are usually representative of studies that focus on the assessment of molecular expression in specific tissues or cell types and can be valuable tools when designing a new experiment or doing quality control and purity assessment of bulk RNA-Seq data.

## Publishing recommendations for RNA-Seq studies in the field of brain barriers

- Include a description of the selected region/tissue/cell type (i.e. capillary endothelial cells from the prefrontal cortex) as well as discarded regions/tissues/cell types (through different purification steps.
- Provide a detailed barriers isolation protocol using a schematic overview that addresses the following points:
  - Relevant details on the tissue source: age, sex and for animal strains and for humans detailed information on the individuals as applicable.
  - Rational for specific brain barriers isolation methodologies used.
  - In depth information on workflow of isolation protocol including precise information on digestion and size-dependent selection steps applied.
  - For bead selection, mention advantages and disadvantages of the used antibodies.
  - Obtained yield of biological material.
  - Test for purity and its result including the rationale for testing for specific contaminants and methodology applied..
- Description of the RNA extraction protocol can be less extensive than that for tissue isolation. However, it should clearly indicate RNA integrity values (or range) as well as quantification.
- Add sequencing specific information, including:
  - Library enrichment—RNAs of interest.
  - Platform.
  - Pairedness.
  - Strandedness.
  - Depth (range).
  - Replicates per group..
- Provide data analysis details:
  - When possible a standardization of a simplified script as supplementary material in all publications.
  - Simplified schematic analytical pipeline with tools and processes.
  - Relevant Quality Control information.
  - Metrics used throughout the paper, such as normalization values, cut-offs (Adjusted P-value and Fold Change for instance). Keep consistent, explain if not.
  - Further criteria for selection should be made clear (possibly in the discussion section, rather than methodology)..
- While referring to protocols in the methods section, point to original paper and make the differences to it clear. Alternatively, include a detailed protocol in the supplementary material.
- At the time of publication make both the raw data, the analyzed data and any relevant metadata publicly available.

### Emerging transcriptomics applications for the field of brain barriers

As mentioned above, the present manuscript has focused on the application of bulk RNA-Seq in the field of brain barriers research. New applications in the field of transcriptomics are however emerging and may thus be of equal significance in the near future.

scRNA-Seq is perhaps the most established and known in brain barriers research. Several studies have used this methodology to great effect allowing to discover a previously unknown diversity of cells in the brain and the brain vasculature, respectively [13, 206]. Single nucleus RNA-Seq (snRNA-Seq) is an alternative method to scRNA-Seq. While it presents a limitation in that only nuclear RNA will be captured and sequenced, it has the advantage of not requiring live dissociated cells. As such, it was used in conjugation with scRNA-Seq to compare adult and embryonic transcriptomic profiles of the ChP in the cases where dissociation failed to produce viable cells [42].

Spatial RNA-Seq is a recent technique that allows to sequence tissue sections in a way that the transcriptomic profiles can be attributed back to different locations in the section, with each location corresponding to a uniquely barcoded spot. Briefly, a tissue section is placed on a special microscopic slide (coated with spatial barcode oligos) that allows to maintain positional information throughout the sequencing process. As such, in conjugation with a previously acquired image of the section taken beforehand, it is possible to attribute specific transcriptomic profiles to regions in the tissue section. At present the resolution of spatial RNA-Seq allows to distinguish about 5000 spots per slide with each spot having a 55 µm diameter and a 100 µm center to center distance between spots. Thus, this technology is not well suited for precise assignment of transcriptomic information to the fine structures of the microvasculature (such as BBB microvessels), but rather to focal areas in the brain and spinal cord allowing to determine regional differences in the transcriptome in the brain and spinal cords in health and disease [207, 208]. As the resolution and the technology improves it has the potential to become a valuable tool for brain barriers research.

As a final note of this section, it is important to point out that none of the mentioned technologies will replace bulk RNA-Seq. Rather, they provide alternative or supplementary technology to bulk RNA-Seq and are accompanied by different strengths and weaknesses. For instance, scRNA-Seq usually requires live, viable, dissociated cells and transcripts of low and medium expression will be underrepresented. The choice of which technology is appropriate should be made based on what the objectives of the study in question are as well as extrinsic factors, such as cost. This decision should be made early on the process and the input of a sequencing facility technician and of a bioinformatician is valuable to the wet-lab researcher.

## Concluding remarks

This manuscript intends to provide a valuable and exhausting source of information for brain barriers researchers and bioinformaticians when planning RNA-Seq analysis in the context of brain barriers research based on the experiences made in the BtRAIN network. Our intention is that this manuscript incites closer interaction between classical brain barriers researchers and bioinformaticians in planning and performing RNA-Seq analysis. We are convinced that considering the issues raised here will allow for future publication of studies providing more specific and accurate information which is prerequisite for data comparison and replication.

## Glossary

- Alignment and Mapping: Alignment is the process of attributing reads to the corresponding place in the genome/transcriptome. Mapping usually refers exclusively to doing this process by aligning the reads to a reference genome while alignment can also refer to a de novo assembly.
- Batch effect: Type of variability not related to the biological question that can obfuscate the results. It tends to have a technical source and it is usually hard to pinpoint. Can be due to different labs, different time days for RNA extraction as well as many other factors.
- BAM file: Binary form of a SAM file (Sequence Alignment/Map), which is the file resulting from the alignment. These files can be sorted by gene name of by genomic coordinates.
- Checksum (i.e. md5sum): algorithm that creates an “hash” (a string of characters) corresponding to a file. Since small changes to the file completely alter this string it can be used to detect corruption of the file during transfer.
- Demultiplexing: in silico process of assigning the reads back to the corresponding sample. During sequencing samples are often multiplexed (pooled and sequenced together) making demultiplexing an essential step.
- Downstream Analysis: Usually refers to the more flexible statistical steps that start with the counts matrix. It includes, amongst others, normalization, differential expression analysis, batch effect correction, multiple testing correction and annotation.
- FASTQ: file type that stores the biological sequence alongside the corresponding PHRED scores. It is usually the file with which the analysis starts.
- Fluorescence-activated cell sorting (FACS): technique that uses flow cytometry to sort a heterogeneous mixture of cells into two or more groups based upon light scattering and fluorescent characteristics.
- Genome Browser: Tool that allows for direct visualization of the reads aligned to a reference genome using the coordinate sorted BAM files.
- Laser capture microdissection (LCM): technique that allows the dissection and isolation of cells or tissue. It uses direct microscopic visualization and a laser. Dissected material can be captured by absorption, ejection, gravity or aspiration.
- Paired or single-end reads: single-end reads are only sequenced from one end of the DNA fragment while paired-end reads are sequenced from both ends. This will have an impact on certain features, for instance, paired-end reads are better suited for the discovery of novel alternative splicing sites.
- PHRED score: Logarithmic quality score for base-calling errors. The higher the score, the less the probability of an error for each base. A PHRED of 3, for instance, corresponds to a 50% probability of a correct call while a PHRED score of 30 corresponds to a 99.9% probability of a correct call.
- Principal component analysis (PCA): statistical method that uses the provided data (for RNA-Seq it usually is the gene expression values) to define unrelated variables. Usually presented as either 2 or 3 dimensional, it can be used in an exploratory fashion to access the sources of variability in the data.
- Quantification: Also sometimes referred to as counting. In this step reads are attributed to features. These features can be genes, transcripts or exons.
- Transcriptome: complete set of transcripts (RNA molecules) in a cell, and their quantity, for a specific developmental stage or physiological condition.
- Transcriptomics: the study of transcriptomes and their functions.
- Raw files: files that have not been processed, usually referring to the fastq files.
- RNA-sequencing (RNA-Seq): high-throughput method for both mapping and quantifying transcriptomes.
- Upstream Analysis: Usually refers to the analysis up until a count file is generated. This includes, but is not restricted to, trimming, alignment, sorting, counting and the initial quality control steps.

## Abbreviations

AQP4: Aquaporin-4
BBB: Blood–brain barrier
BCSFB: Blood–cerebrospinal–fluid barrier
ChP: Choroid plexuses
CNS: Central nervous system
CSF: Cerebrospinal fluid
CVOs: Circumventricular organs
DEG: Differentially expressed genes
dTTP: Deoxythymidine triphosphate
dUTP: Deoxyuridine triphosphate
ERCC: External RNA controls consortium
FACS: Fluorescence-activated cell sorting
FDR: False discovery rate
FFPE: Formalin-fixed paraffin-embedded
FPKM: Fragments per kilobase of transcript per Million mapped reads
gDNA: Genomic DNA
GFAP: Glial fibrillary acidic protein
GM: Gray matter
GO: Gene ontology
IHC: Immunohistochemistry
LCM: Laser capture microdissection
lncRNA: Long non-coding RNA
miRNA: Micro RNA
mRNA: Messenger RNA
NGS: Next-generation sequencing
NVU: Neurovascular unit
PCA: Principal component analysis
PDGFR-β: Platelet-derived growth factor receptor beta
pMBMECs: Primary mouse brain microvascular endothelial cells
QC: Quality control
qPCR: Quantitative real-time polymerase chain reaction
RIN: RNA integrity number
RLE: Relative log expression
RNA-Seq: RNA sequencing
RPKM: Reads per kilobase of transcript per million mapped reads
rRNA: Ribosomal RNA
scRNA-Seq: Single cell RNA sequencing
snRNA-Seq: Single nucleous RNA sequecing
TMM: Trimmed mean of M-values
TPM: Transcripts per million
tRNA: Transfer RNA
UMI: Unique molecular identifiers
WB: Western blot
WM: White matter

## References

[1] Castro Dias M, Mapunda JA, Vladymyrov M, Engelhardt B (2019). Structure and junctional complexes of endothelial, epithelial and glial brain barriers. Int J Mol Sci.

[2] Engelhardt B, Sorokin L (2009). The blood-brain and the blood-cerebrospinal fluid barriers: function and dysfunction. Semin Immunopathol.

[3] Sweeney MD, Zhao Z, Montagne A, Nelson AR, Zlokovic BV (2019). Blood-brain barrier: from physiology to disease and back. Physiol Rev.

[4] Engelhardt B, Vajkoczy P, Weller RO (2017). The movers and shapers in immune privilege of the CNS. Nat Immunol.

[5] Lopes Pinheiro MA, Kooij G, Mizee MR, Kamermans A, Enzmann G, Lyck R (2016). Immune cell trafficking across the barriers of the central nervous system in multiple sclerosis and stroke. Biochim Biophys Acta.

[6] Marchetti L, Engelhardt B (2020). Immune cell trafficking across the blood-brain barrier in the absence and presence of neuroinflammation. Vasc Biol.

[7] Yasuda K, Cline C, Vogel P, Onciu M, Fatima S, Sorrentino BP (2013). Drug transporters on arachnoid barrier cells contribute to the blood-cerebrospinal fluid barrier. Drug Metabol Dispos.

[8] Ballabh P, Braun A, Nedergaard M (2004). The blood-brain barrier: an overview: structure, regulation, and clinical implications. Neurobiol Dis.

[9] Cardoso FL, Brites D, Brito MA (2010). Looking at the blood-brain barrier: molecular anatomy and possible investigation approaches. Brain Res Rev.

[10] Qaiser MZ, Dolman DEM, Begley DJ, Abbott NJ, Cazacu-Davidescu M, Corol DI (2017). Uptake and metabolism of sulphated steroids by the blood-brain barrier in the adult male rat. J Neurochem.

[11] Serlin Y, Shelef I, Knyazer B, Friedman A (2015). Anatomy and physiology of the blood-brain barrier. Semin Cell Dev Biol.

[12] Daneman R, Zhou L, Agalliu D, Cahoy JD, Kaushal A, Barres BA (2010). The mouse blood–brain barrier transcriptome: a new resource for understanding the development and function of brain endothelial cells. PLoS ONE.

[13] Vanlandewijck M, He L, Mae MA, Andrae J, Ando K, Del Gaudio F (2018). A molecular atlas of cell types and zonation in the brain vasculature. Nature.

[14] Munji RN, Soung AL, Weiner GA, Sohet F, Semple BD, Trivedi A (2019). Profiling the mouse brain endothelial transcriptome in health and disease models reveals a core blood-brain barrier dysfunction module. Nat Neurosci.

[15] Ozsolak F, Milos PM (2011). RNA sequencing: advances, challenges and opportunities. Nat Rev Genet.

[16] Behjati S, Tarpey PS (2013). What is next generation sequencing?. Arch Dis Childh Educ Pract Ed.

[17] Metzker ML (2010). Sequencing technologies—the next generation. Nat Rev Genet.

[18] Wilhelm I, Nyul-Toth A, Suciu M, Hermenean A, Krizbai IA (2016). Heterogeneity of the blood-brain barrier. Tissue Barr.

[19] Borowsky IW, Collins RC (1989). Metabolic anatomy of brain: a comparison of regional capillary density, glucose metabolism, and enzyme activities. J Comp Neurol..

[20] Gross PM, Sposito NM, Pettersen SE, Fenstermacher JD (1986). Differences in function and structure of the capillary endothelium in gray matter, white matter and a circumventricular organ of rat brain. Blood vessels..

[21] Cavaglia M, Dombrowski SM, Drazba J, Vasanji A, Bokesch PM, Janigro D (2001). Regional variation in brain capillary density and vascular response to ischemia. Brain Res.

[22] Nyul-Toth A, Suciu M, Molnar J, Fazakas C, Hasko J, Herman H (2016). Differences in the molecular structure of the blood-brain barrier in the cerebral cortex and white matter: an in silico, in vitro, and ex vivo study. Am J Physiol Heart Circ Physiol..

[23] Bachoo RM, Kim RS, Ligon KL, Maher EA, Brennan C, Billings N (2004). Molecular diversity of astrocytes with implications for neurological disorders. Proc Natl Acad Sci USA.

[24] Sofroniew MV, Vinters HV (2010). Astrocytes: biology and pathology. Acta Neuropathol.

[25] Miyata S (2015). New aspects in fenestrated capillary and tissue dynamics in the sensory circumventricular organs of adult brains. Front Neurosci..

[26] Saubamea B, Cochois-Guegan V, Cisternino S, Scherrmann JM (2012). Heterogeneity in the rat brain vasculature revealed by quantitative confocal analysis of endothelial barrier antigen and P-glycoprotein expression. J Cereb Blood Flow Metab.

[27] Yamazaki T, Mukouyama Y (2018). Tissue specific origin, development, and pathological perspectives of pericytes. Front Cardiovasc Med.

[28] McConnell HL, Kersch CN, Woltjer RL, Neuwelt EA (2017). The translational significance of the neurovascular unit. J Biol Chem.

[29] Ge S, Song L, Pachter JS (2005). Where is the blood-brain barrier really?. J Neurosci Res..

[30] Bechmann I, Galea I, Perry VH (2007). What is the blood-brain barrier (not)?. Trends Immunol.

[31] Dalkara T, Gursoy-Ozdemir Y, Yemisci M (2011). Brain microvascular pericytes in health and disease. Acta Neuropathol.

[32] Dahl E (1973). The fine structure of intracerebral vessels. Z Zellforsch Mikrosk Anat..

[33] Simionescu M, Simionescu N, Palade GE (1976). Segmental differentiations of cell junctions in the vascular endothelium. Arteries and veins. J Cell Biol..

[34] Mayhan WG, Heistad DD (1985). Permeability of blood–brain barrier to various sized molecules. Am J Physiol.

[35] Virgintino D, Robertson D, Errede M, Benagiano V, Girolamo F, Maiorano E (2002). Expression of P-glycoprotein in human cerebral cortex microvessels. J Histochem Cytochem.

[36] Golden PL, Pardridge WM (2000). Brain microvascular P-glycoprotein and a revised model of multidrug resistance in brain. Cell Mol Neurobiol.

[37] Kalaria RN, Sromek SM, Grahovac I, Harik SI (1992). Transferrin receptors of rat and human brain and cerebral microvessels and their status in Alzheimer’s disease. Brain Res.

[38] Wolff JE, Belloni-Olivi L, Bressler JP, Goldstein GW (1992). Gamma-glutamyl transpeptidase activity in brain microvessels exhibits regional heterogeneity. J Neurochem.

[39] Hanigan MH, Frierson HF (1996). Immunohistochemical detection of gamma-glutamyl transpeptidase in normal human tissue. J Histochem Cytochem.

[40] Sheikov N, McDannold N, Jolesz F, Zhang YZ, Tam K, Hynynen K (2006). Brain arterioles show more active vesicular transport of blood-borne tracer molecules than capillaries and venules after focused ultrasound-evoked opening of the blood-brain barrier. Ultrasound Med Biol.

[41] Lun MP, Johnson MB, Broadbelt KG, Watanabe M, Kang YJ, Chau KF (2015). Spatially heterogeneous choroid plexus transcriptomes encode positional identity and contribute to regional CSF production. J Neurosci.

[42] Dani N, Herbst RH, Habib N, Head J, Dionne D, Nguyen L (2019). A cellular and spatial map of the choroid plexus across brain ventricles and ages. bioRxiv..

[43] Sohet F, Daneman R (2013). Genetic mouse models to study blood–brain barrier development and function. Fluids Barriers CNS.

[44] Johnson BA, Coutts M, Vo HM, Hao X, Fatima N, Rivera MJ (2018). Accurate, strong, and stable reporting of choroid plexus epithelial cells in transgenic mice using a human transthyretin BAC. Fluids Barriers CNS..

[45] Lobas MA, Helsper L, Vernon CG, Schreiner D, Zhang Y, Holtzman MJ (2012). Molecular heterogeneity in the choroid plexus epithelium: the 22-member gamma-protocadherin family is differentially expressed, apically localized, and implicated in CSF regulation. J Neurochem.

[46] Becher B, Waisman A, Lu LF (2019). Cre-lox: target sensitivity matters. Immunity.

[47] Assmann JC, Korbelin J, Schwaninger M (2016). Genetic manipulation of brain endothelial cells in vivo. Biochim Biophys Acta.

[48] Brendel K, Meezan E, Carlson EC (1974). Isolated brain microvessels: a purified, metabolically active preparation from bovine cerebral cortex. Science.

[49] DeBault LE, Kahn LE, Frommes SP, Cancilla PA (1979). Cerebral microvessels and derived cells in tissue culture: isolation and preliminary characterization. Vitro..

[50] Pardridge WM, Eisenberg J, Yamada T (1985). Rapid sequestration and degradation of somatostatin analogues by isolated brain microvessels. J Neurochem.

[51] Scott PA, Bicknell R (1993). The isolation and culture of microvascular endothelium. J Cell Sci.

[52] Wu Z, Hofman FM, Zlokovic BV (2003). A simple method for isolation and characterization of mouse brain microvascular endothelial cells. J Neurosci Methods.

[53] Hewett PW, Murray JC (1993). Human lung microvessel endothelial cells: isolation, culture, and characterization. Microvasc Res.

[54] van Beijnum JR, Rousch M, Castermans K, van der Linden E, Griffioen AW (2008). Isolation of endothelial cells from fresh tissues. Nat Protoc.

[55] Betz AL, Csejtey J, Goldstein GW (1979). Hexose transport and phosphorylation by capillaries isolated from rat brain. Am J Physiol.

[56] Bernas MJ, Cardoso FL, Daley SK, Weinand ME, Campos AR, Ferreira AJ (2010). Establishment of primary cultures of human brain microvascular endothelial cells to provide an in vitro cellular model of the blood-brain barrier. Nat Protoc.

[57] Hartz AMS, Schulz JA, Sokola BS, Edelmann SE, Shen AN, Rempe RG (2018). Isolation of cerebral capillaries from fresh human brain tissue. JoVE.

[58] Coisne C, Dehouck L, Faveeuw C, Delplace Y, Miller F, Landry C (2005). Mouse syngenic in vitro blood-brain barrier model: a new tool to examine inflammatory events in cerebral endothelium. Lab Invest.

[59] Yousif S, Marie-Claire C, Roux F, Scherrmann JM, Decleves X (2007). Expression of drug transporters at the blood-brain barrier using an optimized isolated rat brain microvessel strategy. Brain Res.

[60] Itoh Y, Suzuki N (2012). Control of brain capillary blood flow. J Cereb Blood Flow Metab.

[61] Vajkoczy P, Laschinger M, Engelhardt B (2001). Alpha4-integrin-VCAM-1 binding mediates G protein-independent capture of encephalitogenic T cell blasts to CNS white matter microvessels. J Clin Invest..

[62] Xue S, Gong H, Jiang T, Luo W, Meng Y, Liu Q (2014). Indian-ink perfusion based method for reconstructing continuous vascular networks in whole mouse brain. PLoS ONE.

[63] Sekiguchi Y, Masamoto K, Takuwa H, Kawaguchi H, Kanno I, Ito H (2013). Measuring the vascular diameter of brain surface and parenchymal arteries in awake mouse. Adv Exp Med Biol.

[64] Qian B, Rudy RF, Cai T, Du R (2018). Cerebral artery diameter in inbred mice varies as a function of strain. Front Neuroanat..

[65] Jensen-Urstad K, Jensen-Urstad M, Johansson J (1999). Carotid artery diameter correlates with risk factors for cardiovascular disease in a population of 55-year-old subjects. Stroke.

[66] Seidman MD, Khan MJ, Dolan DF, Quirk WS (1996). Age-related differences in cochlear microcirculation and auditory brain stem response. Arch Otolaryngol..

[67] Demeule M, Labelle M, Regina A, Berthelet F, Beliveau R (2001). Isolation of endothelial cells from brain, lung, and kidney: expression of the multidrug resistance P-glycoprotein isoforms. Biochem Biophys Res Commun.

[68] Vissapragada R, Contreras MA, da Silva CG, Kumar VA, Ochoa A, Vasudevan A (2014). Bidirectional crosstalk between periventricular endothelial cells and neural progenitor cells promotes the formation of a neurovascular unit. Brain Res.

[69] Lyck R, Ruderisch N, Moll AG, Steiner O, Cohen CD, Engelhardt B (2009). Culture-induced changes in blood-brain barrier transcriptome: implications for amino-acid transporters in vivo. J Cereb Blood Flow Metab.

[70] Miebach S, Grau S, Hummel V, Rieckmann P, Tonn JC, Goldbrunner RH (2006). Isolation and culture of microvascular endothelial cells from gliomas of different WHO grades. J Neurooncol.

[71] Bagley RG, Rouleau C, Morgenbesser SD, Weber W, Cook BP, Shankara S (2006). Pericytes from human non-small cell lung carcinomas: an attractive target for anti-angiogenic therapy. Microvasc Res.

[72] Feldmann M, Pathipati P, Sheldon RA, Jiang X, Ferriero DM. Isolating astrocytes and neurons sequentially from postnatal murine brains with a magnetic cell separation technique. 2014. 2014.

[73] Wilson CL, Stephenson SE, Higuero JP, Feghali-Bostwick C, Hung CF, Schnapp LM (2018). Characterization of human PDGFR-β-positive pericytes from IPF and non-IPF lungs. Am J Physiol Lung Cell Mol Physiol.

[74] Abbott NJ, Rönnbäck L, Hansson E (2006). Astrocyte–endothelial interactions at the blood–brain barrier. Nat Rev Neurosci.

[75] Vandenhaute E, Dehouck L, Boucau MC, Sevin E, Uzbekov R, Tardivel M (2011). Modelling the neurovascular unit and the blood-brain barrier with the unique function of pericytes. Curr Neurovasc Res..

[76] He L, Vanlandewijck M, Mae MA, Andrae J, Ando K, Del Gaudio F (2018). Single-cell RNA sequencing of mouse brain and lung vascular and vessel-associated cell types. Sci Data..

[77] Dong QG, Bernasconi S, Lostaglio S, De Calmanovici RW, Martin-Padura I, Breviario F (1997). A general strategy for isolation of endothelial cells from murine tissues. Characterization of two endothelial cell lines from the murine lung and subcutaneous sponge implants. Arterioscler Thromb Vasc Biol..

[78] Marelli-Berg FM, Peek E, Lidington EA, Stauss HJ, Lechler RI (2000). Isolation of endothelial cells from murine tissue. J Immunol Methods.

[79] Castro Dias M, Coisne C, Lazarevic I, Baden P, Hata M, Iwamoto N (2019). Claudin-3-deficient C57BL/6 J mice display intact brain barriers. Sci Rep..

[80] Czupalla CJ, Yousef H, Wyss-Coray T, Butcher EC (2018). Collagenase-based single cell isolation of primary murine brain endothelial cells using flow cytometry. Bio Protoc..

[81] Yousef H, Czupalla CJ, Lee D, Butcher EC, Wyss-Coray T (2018). Papain-based single cell isolation of primary murine brain endothelial cells using flow cytometry. Bio Protoc..

[82] Crouch EE, Doetsch F (2018). FACS isolation of endothelial cells and pericytes from mouse brain microregions. Nat Protoc.

[83] Armulik A, Genove G, Mae M, Nisancioglu MH, Wallgard E, Niaudet C (2010). Pericytes regulate the blood–brain barrier. Nature.

[84] Novak A, Guo C, Yang W, Nagy A, Lobe CG (2000). Z/EG, a double reporter mouse line that expresses enhanced green fluorescent protein upon Cre-mediated excision. Genesis..

[85] Cossarizza A, Chang HD, Radbruch A, Akdis M, Andra I, Annunziato F (2017). Guidelines for the use of flow cytometry and cell sorting in immunological studies. Eur J Immunol.

[86] Hermkens DMA, Stam OCG, de Wit NM, Fontijn RD, Jongejan A, Moerland PD (2019). Profiling the unique protective properties of intracranial arterial endothelial cells. Acta Neuropathol Commun.

[87] Cunnea P, McMahon J, O’Connell E, Mashayekhi K, Fitzgerald U, McQuaid S (2010). Gene expression analysis of the microvascular compartment in multiple sclerosis using laser microdissected blood vessels. Acta Neuropathol.

[88] Ball HJ, McParland B, Driussi C, Hunt NH (2002). Isolating vessels from the mouse brain for gene expression analysis using laser capture microdissection. Brain Res Brain Res Protoc..

[89] Mojsilovic-Petrovic J, Nesic M, Pen A, Zhang W, Stanimirovic D (2004). Development of rapid staining protocols for laser-capture microdissection of brain vessels from human and rat coupled to gene expression analyses. J Neurosci Methods.

[90] Macdonald JA, Murugesan N, Pachter JS (2008). Validation of immuno-laser capture microdissection coupled with quantitative RT-PCR to probe blood-brain barrier gene expression in situ. J Neurosci Methods.

[91] Risau W, Engelhardt B, Wekerle H (1990). Immune function of the blood-brain barrier: incomplete presentation of protein (auto-)antigens by rat brain microvascular endothelium in vitro. J Cell Biol.

[92] Stone NL, England TJ, O’Sullivan SE (2019). A Novel Transwell Blood Brain Barrier Model Using Primary Human Cells. Front Cell Neurosci..

[93] Paradis A, Leblanc D, Dumais N (2016). Optimization of an in vitro human blood-brain barrier model: application to blood monocyte transmigration assays. MethodsX..

[94] Ito R, Umehara K, Suzuki S, Kitamura K, Nunoya KI, Yamaura Y (2019). A human immortalized cell-based blood–brain barrier triculture model: development and characterization as a promising tool for drug-brain permeability studies. Mol Pharm.

[95] Adriani G, Ma D, Pavesi A, Goh EL, Kamm RD (2015). Modeling the blood–brain barrier in a 3D triple co-culture microfluidic system. Conf Proc IEEE Eng Med Biol Soc..

[96] Idris F, HannaMuharram S, Zaini Z, Diah S (2018). Establishment of murine <em> in vitro </em> blood-brain barrier models using immortalized cell lines: co-cultures of brain endothelial cells, astrocytes, and neurons. bioRxiv..

[97] Kalari KR, Thompson KJ, Nair AA, Tang X, Bockol MA, Jhawar N (2016). BBBomics-human blood brain barrier transcriptomics hub. Front Neurosci.

[98] Cecchelli R, Aday S, Sevin E, Almeida C, Culot M, Dehouck L (2014). A stable and reproducible human blood–brain barrier model derived from hematopoietic stem cells. PLoS ONE.

[99] Workman MJ, Svendsen CN (2020). Recent advances in human iPSC-derived models of the blood–brain barrier. Fluids Barriers CNS..

[100] Qian T, Maguire SE, Canfield SG, Bao X, Olson WR, Shusta EV (2017). Directed differentiation of human pluripotent stem cells to blood-brain barrier endothelial cells. Sci Adv.

[101] Lippmann ES, Azarin SM, Kay JE, Nessler RA, Wilson HK, Al-Ahmad A (2012). Derivation of blood-brain barrier endothelial cells from human pluripotent stem cells. Nat Biotechnol.

[102] Stebbins MJ, Wilson HK, Canfield SG, Qian T, Palecek SP, Shusta EV (2016). Differentiation and characterization of human pluripotent stem cell-derived brain microvascular endothelial cells. Methods.

[103] Ghersi-Egea J-F, Strazielle N, Catala M, Silva-Vargas V, Doetsch F, Engelhardt B (2018). Molecular anatomy and functions of the choroidal blood-cerebrospinal fluid barrier in health and disease. Acta Neuropathol.

[104] Kim S, Hwang Y, Lee D, Webster MJ (2016). Transcriptome sequencing of the choroid plexus in schizophrenia. Transl Psychiatry..

[105] Stopa EG, Tanis KQ, Miller MC, Nikonova EV, Podtelezhnikov AA, Finney EM (2018). Comparative transcriptomics of choroid plexus in Alzheimer’s disease, frontotemporal dementia and Huntington’s disease: implications for CSF homeostasis. Fluids Barriers CNS..

[106] Hasselblatt M, Bohm C, Tatenhorst L, Dinh V, Newrzella D, Keyvani K (2006). Identification of novel diagnostic markers for choroid plexus tumors: a microarray-based approach. Am J Surg Pathol.

[107] Hasselblatt M, Mertsch S, Koos B, Riesmeier B, Stegemann H, Jeibmann A (2009). TWIST-1 is overexpressed in neoplastic choroid plexus epithelial cells and promotes proliferation and invasion. Cancer Res.

[108] Bergen AA, Kaing S, ten Brink JB, Netherlands Brain B, Gorgels TG, Janssen SF (2015). Gene expression and functional annotation of human choroid plexus epithelium failure in Alzheimer’s disease. BMC Genomics..

[109] Strazielle N, Ghersi-Egea JF (1999). Demonstration of a coupled metabolism-efflux process at the choroid plexus as a mechanism of brain protection toward xenobiotics. J Neurosci.

[110] Lazarevic I, Engelhardt B (2016). Modeling immune functions of the mouse blood-cerebrospinal fluid barrier in vitro: primary rather than immortalized mouse choroid plexus epithelial cells are suited to study immune cell migration across this brain barrier. Fluids Barriers CNS..

[111] Sanders-Bush E, Breeding M (1991). Choroid plexus epithelial cells in primary culture: a model of 5HT1C receptor activation by hallucinogenic drugs. Psychopharmacology.

[112] Wollack JB, Makori B, Ahlawat S, Koneru R, Picinich SC, Smith A (2008). Characterization of folate uptake by choroid plexus epithelial cells in a rat primary culture model. J Neurochem.

[113] Van Hove H, Martens L, Scheyltjens I, De Vlaminck K, Pombo Antunes AR, De Prijck S (2019). A single-cell atlas of mouse brain macrophages reveals unique transcriptional identities shaped by ontogeny and tissue environment. Nat Neurosci.

[114] Mobasheri A, Marples D, Young IS, Floyd RV, Moskaluk CA, Frigeri A (2007). Distribution of the AQP4 water channel in normal human tissues: protein and tissue microarrays reveal expression in several new anatomical locations, including the prostate gland and seminal vesicles. Channels.

[115] Speake T, Freeman LJ, Brown PD (2003). Expression of aquaporin 1 and aquaporin 4 water channels in rat choroid plexus. Biochim Biophys Acta.

[116] Nielsen S, Nagelhus EA, Amiry-Moghaddam M, Bourque C, Agre P, Ottersen OP (1997). Specialized membrane domains for water transport in glial cells: high-resolution immunogold cytochemistry of aquaporin-4 in rat brain. J Neurosci.

[117] Skalli O, Pelte MF, Peclet MC, Gabbiani G, Gugliotta P, Bussolati G (1989). Alpha-smooth muscle actin, a differentiation marker of smooth muscle cells, is present in microfilamentous bundles of pericytes. J Histochem Cytochem.

[118] Alarcon-Martinez L, Yilmaz-Ozcan S, Yemisci M, Schallek J, Kilic K, Can A (2018). Capillary pericytes express alpha-smooth muscle actin, which requires prevention of filamentous-actin depolymerization for detection. eLife..

[119] Skalli O, Ropraz P, Trzeciak A, Benzonana G, Gillessen D, Gabbiani G (1986). A monoclonal antibody against alpha-smooth muscle actin: a new probe for smooth muscle differentiation. J Cell Biol.

[120] Newman PJ, Berndt MC, Gorski J, White GC, Lyman S, Paddock C (1990). PECAM-1 (CD31) cloning and relation to adhesion molecules of the immunoglobulin gene superfamily. Science.

[121] Brown H, Hien TT, Day N, Mai NT, Chuong LV, Chau TT (1999). Evidence of blood-brain barrier dysfunction in human cerebral malaria. Neuropathol Appl Neurobiol.

[122] Murugesan N, Paul D, Lemire Y, Shrestha B, Ge S, Pachter JS (2012). Active induction of experimental autoimmune encephalomyelitis by MOG35-55 peptide immunization is associated with differential responses in separate compartments of the choroid plexus. Fluids Barriers CNS..

[123] Stockinger H, Gadd SJ, Eher R, Majdic O, Schreiber W, Kasinrerk W (1990). Molecular characterization and functional analysis of the leukocyte surface protein CD31. J Immunol..

[124] Correale J, Villa A (2009). Cellular elements of the blood–brain barrier. Neurochem Res.

[125] Alva JA, Zovein AC, Monvoisin A, Murphy T, Salazar A, Harvey NL (2006). VE-Cadherin-Cre-recombinase transgenic mouse: a tool for lineage analysis and gene deletion in endothelial cells. Dev Dyn.

[126] Liebner S, Kniesel U, Kalbacher H, Wolburg H (2000). Correlation of tight junction morphology with the expression of tight junction proteins in blood-brain barrier endothelial cells. Eur J Cell Biol.

[127] Lippoldt A, Liebner S, Andbjer B, Kalbacher H, Wolburg H, Haller H (2000). Organization of choroid plexus epithelial and endothelial cell tight junctions and regulation of claudin-1, -2 and -5 expression by protein kinase C. NeuroReport.

[128] Wolburg H, Wolburg-Buchholz K, Liebner S, Engelhardt B (2001). Claudin-1, claudin-2 and claudin-11 are present in tight junctions of choroid plexus epithelium of the mouse. Neurosci Lett.

[129] Pfeiffer F, Schafer J, Lyck R, Makrides V, Brunner S, Schaeren-Wiemers N (2011). Claudin-1 induced sealing of blood-brain barrier tight junctions ameliorates chronic experimental autoimmune encephalomyelitis. Acta Neuropathol.

[130] Wolburg H, Wolburg-Buchholz K, Kraus J, Rascher-Eggstein G, Liebner S, Hamm S (2003). Localization of claudin-3 in tight junctions of the blood-brain barrier is selectively lost during experimental autoimmune encephalomyelitis and human glioblastoma multiforme. Acta Neuropathol.

[131] Nitta T, Hata M, Gotoh S, Seo Y, Sasaki H, Hashimoto N (2003). Size-selective loosening of the blood-brain barrier in claudin-5-deficient mice. J Cell Biol.

[132] Yamamoto M, Ramirez SH, Sato S, Kiyota T, Cerny RL, Kaibuchi K (2008). Phosphorylation of claudin-5 and occludin by rho kinase in brain endothelial cells. Am J Pathol.

[133] Ulfig N, Briese M, Bohl J (2002). Expression of Oligodendrocyte-Specific Protein/Claudin-11 in the Human Fetal Forebrain. Neuroembryol Aging.

[134] Tiwari-Woodruff SK, Buznikov AG, Vu TQ, Micevych PE, Chen K, Kornblum HI (2001). OSP/claudin-11 forms a complex with a novel member of the tetraspanin super family and beta1 integrin and regulates proliferation and migration of oligodendrocytes. J Cell Biol.

[135] Riva MA, Mocchetti I (1991). Developmental expression of the basic fibroblast growth factor gene in rat brain. Brain Res Dev Brain Res.

[136] Bignami A, Eng LF, Dahl D, Uyeda CT (1972). Localization of the glial fibrillary acidic protein in astrocytes by immunofluorescence. Brain Res.

[137] Duan S, Anderson CM, Stein BA, Swanson RA (1999). Glutamate induces rapid upregulation of astrocyte glutamate transport and cell-surface expression of GLAST. J Neurosci.

[138] Defazio G, Trojano M, Ribatti D, Nico B, Giorelli M, De Salvia R (1998). ICAM 1 expression and fluid phase endocytosis of cultured brain microvascular endothelial cells following exposure to interferon beta-1a and TNFalpha. J Neuroimmunol.

[139] Sobel RA, Mitchell ME, Fondren G (1990). Intercellular adhesion molecule-1 (ICAM-1) in cellular immune reactions in the human central nervous system. Am J Pathol.

[140] Verbeek MM, Otte-Holler I, Wesseling P, Ruiter DJ, de Waal RM (1994). Induction of alpha-smooth muscle actin expression in cultured human brain pericytes by transforming growth factor-beta 1. Am J Pathol.

[141] Rothlein R, Dustin ML, Marlin SD, Springer TA (1986). A human intercellular adhesion molecule (ICAM-1) distinct from LFA-1. J Immunol..

[142] Chang CH, Hong SC, Hughes CC, Janeway CA, Flavell RA (1995). CIITA activates the expression of MHC class II genes in mouse T cells. Int Immunol.

[143] Daneman R, Zhou L, Kebede AA, Barres BA (2010). Pericytes are required for blood-brain barrier integrity during embryogenesis. Nature.

[144] Alghamdi B, Fern R (2015). Phenotype overlap in glial cell populations: astroglia, oligodendroglia and NG-2(+) cells. Front Neuroanat.

[145] Breiteneder-Geleff S, Matsui K, Soleiman A, Meraner P, Poczewski H, Kalt R (1997). Podoplanin, novel 43-kd membrane protein of glomerular epithelial cells, is down-regulated in puromycin nephrosis. Am J Pathol.

[146] Tomooka M, Kaji C, Kojima H, Sawa Y (2013). Distribution of podoplanin-expressing cells in the mouse nervous systems. Acta Histochem Cytochem.

[147] Wong D, Dorovini-Zis K (1995). Expression of vascular cell adhesion molecule-1 (VCAM-1) by human brain microvessel endothelial cells in primary culture. Microvasc Res.

[148] Jiao H, Wang Z, Liu Y, Wang P, Xue Y (2011). Specific role of tight junction proteins claudin-5, occludin, and ZO-1 of the blood-brain barrier in a focal cerebral ischemic insult. J Mol Neurosci.

[149] Krause D, Mischeck U, Galla HJ, Dermietzel R (1991). Correlation of zonula occludens ZO-1 antigen expression and transendothelial resistance in porcine and rat cultured cerebral endothelial cells. Neurosci Lett.

[150] Tan SC, Yiap BC (2009). DNA, RNA, and protein extraction: the past and the present. J Biomed Biotechnol..

[151] Tavares L, Alves PM, Ferreira RB, Santos CN (2011). Comparison of different methods for DNA-free RNA isolation from SK-N-MC neuroblastoma. BMC Res Notes..

[152] Chatterjee A, Ahn A, Rodger EJ, Stockwell PA, Eccles MR (2018). A Guide for Designing and Analyzing RNA-Seq Data. Methods Mol Biol.

[153] Andreasson A, Kiss NB, Juhlin CC, Höög A (2013). Long-term storage of endocrine tissues at − 80 °C does not adversely affect rna quality or overall histomorphology. Biopreserv Biobank.

[154] Abramovitz M, Ordanic-Kodani M, Wang Y, Li Z, Catzavelos C, Bouzyk M (2008). Optimization of RNA extraction from FFPE tissues for expression profiling in the DASL assay. Biotechniques.

[155] Schroeder A, Mueller O, Stocker S, Salowsky R, Leiber M, Gassmann M (2006). The RIN: an RNA I ntegrity number for assigning integrity values to RNA measurements. BMC Mol Biol.

[156] Esteve-Codina A, Arpi O, Martinez-Garcia M, Pineda E, Mallo M, Gut M (2017). A comparison of RNA-Seq results from paired formalin-fixed paraffin-embedded and fresh-frozen glioblastoma tissue samples. PLoS ONE.

[157] Li J, Fu C, Speed TP, Wang W, Symmans WF (2018). Accurate RNA sequencing from formalin-fixed cancer tissue to represent high-quality transcriptome from frozen tissue. JCO Precis Oncol.

[158] Biegel D, Spencer DD, Pachter JS (1995). Isolation and culture of human brain microvessel endothelial cells for the study of blood–brain barrier properties in vitro. Brain Res.

[159] Paul G, Ozen I, Christophersen NS, Reinbothe T, Bengzon J, Visse E (2012). The adult human brain harbors multipotent perivascular mesenchymal stem cells. PLoS ONE.

[160] Shawahna R, Uchida Y, Decleves X, Ohtsuki S, Yousif S, Dauchy S (2011). Transcriptomic and quantitative proteomic analysis of transporters and drug metabolizing enzymes in freshly isolated human brain microvessels. Mol Pharm.

[161] Spaethling JM, Na YJ, Lee J, Ulyanova AV, Baltuch GH, Bell TJ (2017). Primary cell culture of live neurosurgically resected aged adult human brain cells and single cell transcriptomics. Cell Rep..

[162] Palladino SP, Helton ES, Jain P, Dong C, Crowley MR, Crossman DK (2017). The human blood-nerve barrier transcriptome. Sci Rep..

[163] Dauchy S, Miller F, Couraud PO, Weaver RJ, Weksler B, Romero IA (2009). Expression and transcriptional regulation of ABC transporters and cytochromes P450 in hCMEC/D3 human cerebral microvascular endothelial cells. Biochem Pharmacol.

[164] Barton AJ, Pearson RC, Najlerahim A, Harrison PJ (1993). Pre- and postmortem influences on brain RNA. J Neurochem.

[165] Gallego Romero I, Pai AA, Tung J, Gilad Y (2014). RNA-seq: impact of RNA degradation on transcript quantification. BMC Biol.

[166] Sutherland GT, Sheedy D, Kril JJ (2014). Using autopsy brain tissue to study alcohol-related brain damage in the genomic age. Alcohol Clin Exp Res.

[167] Johnson SA, Morgan DG, Finch CE (1986). Extensive postmortem stability of RNA from rat and human brain. J Neurosci Res.

[168] Cummings TJ, Strum JC, Yoon LW, Szymanski MH, Hulette CM (2001). Recovery and expression of messenger RNA from postmortem human brain tissue. Mod Pathol.

[169] Williams AG, Thomas S, Wyman SK, Holloway AK (2014). RNA-seq data: challenges in and recommendations for experimental design and analysis. Curr Protoc Hum Genet.

[170] Ching T, Huang S, Garmire LX (2014). Power analysis and sample size estimation for RNA-Seq differential expression. RNA.

[171] Liu Y, Zhou J, White KP (2014). RNA-seq differential expression studies: more sequence or more replication?. Bioinformatics.

[172] Baker SC, Bauer SR, Beyer RP, Brenton JD, Bromley B, Burrill J (2005). The external RNA controls consortium: a progress report. Nat Methods.

[173] Hampl R, Bicikova M, Sosvorova L (2015). Hormones and the blood-brain barrier. Hormone Mol Biol Clin Investig.

[174] Conesa A, Madrigal P, Tarazona S, Gomez-Cabrero D, Cervera A, McPherson A (2016). A survey of best practices for RNA-seq data analysis. Genome Biol.

[175] Hansen KD, Wu Z, Irizarry RA, Leek JT (2011). Sequencing technology does not eliminate biological variability. Nat Biotechnol.

[176] Schurch NJ, Schofield P, Gierlinski M, Cole C, Sherstnev A, Singh V (2016). How many biological replicates are needed in an RNA-seq experiment and which differential expression tool should you use?. RNA.

[177] Zhao S, Zhang Y, Gordon W, Quan J, Xi H, Du S (2015). Comparison of stranded and non-stranded RNA-seq transcriptome profiling and investigation of gene overlap. BMC Genomics..

[178] Nold-Petry CA, Lo CY, Rudloff I, Elgass KD, Li S, Gantier MP (2015). IL-37 requires the receptors IL-18Ralpha and IL-1R8 (SIGIRR) to carry out its multifaceted anti-inflammatory program upon innate signal transduction. Nat Immunol.

[179] Tushev G, Glock C, Heumuller M, Biever A, Jovanovic M, Schuman EM (2018). Alternative 3′ UTRs modify the localization, regulatory potential, stability, and plasticity of mRNAs in neuronal compartments. Neuron.

[180] Tosches MA, Yamawaki TM, Naumann RK, Jacobi AA, Tushev G, Laurent G (2018). Evolution of pallium, hippocampus, and cortical cell types revealed by single-cell transcriptomics in reptiles. Science.

[181] Ma F, Fuqua BK, Hasin Y, Yukhtman C, Vulpe CD, Lusis AJ (2019). A comparison between whole transcript and 3′ RNA sequencing methods using Kapa and Lexogen library preparation methods. BMC Genomics..

[182] Toyama K, Spin JM, Tsao PS (2017). Role of microRNAs on blood brain barrier dysfunction in vascular cognitive impairment. Curr Drug Deliv.

[183] Xi T, Jin F, Zhu Y, Wang J, Tang L, Wang Y (2018). miR-27a-3p protects against blood-brain barrier disruption and brain injury after intracerebral hemorrhage by targeting endothelial aquaporin-11. J Biol Chem.

[184] Grada A, Weinbrecht K (2013). Next-generation sequencing: methodology and application. J Invest Dermatol..

[185] Berglund EC, Kiialainen A, Syvanen AC (2011). Next-generation sequencing technologies and applications for human genetic history and forensics. Investig Genet..

[186] Quail MA, Smith M, Coupland P, Otto TD, Harris SR, Connor TR (2012). A tale of three next generation sequencing platforms: comparison of Ion Torrent, Pacific Biosciences and Illumina MiSeq sequencers. BMC Genomics..

[187] Lahens NF, Ricciotti E, Smirnova O, Toorens E, Kim EJ, Baruzzo G (2017). A comparison of Illumina and Ion Torrent sequencing platforms in the context of differential gene expression. BMC Genomics..

[188] Rhoads A, Au KF (2015). PacBio sequencing and its applications. Genom Proteom Bioinf.

[189] Jain M, Koren S, Miga KH, Quick J, Rand AC, Sasani TA (2018). Nanopore sequencing and assembly of a human genome with ultra-long reads. Nat Biotechnol.

[190] Quinn TP, Erb I, Richardson MF, Crowley TM (2018). Understanding sequencing data as compositions: an outlook and review. Bioinformatics.

[191] Andrews S. FastQC: a quality control tool for high throughput sequence data. 2010.

[192] Robinson JT, Thorvaldsdóttir H, Winckler W, Guttman M, Lander ES, Getz G (2011). Integrative genomics viewer. Nat Biotechnol.

[193] Thorvaldsdottir H, Robinson JT, Mesirov JP (2013). Integrative Genomics Viewer (IGV): high-performance genomics data visualization and exploration. Brief Bioinf.

[194] Wagner GP, Kin K, Lynch VJ (2012). Measurement of mRNA abundance using RNA-seq data: rPKM measure is inconsistent among samples. Theory Biosci.

[195] Robinson MD, McCarthy DJ, Smyth GK (2010). edgeR: a Bioconductor package for differential expression analysis of digital gene expression data. Bioinformatics.

[196] Love MI, Huber W, Anders S (2014). Moderated estimation of fold change and dispersion for RNA-seq data with DESeq2. Genome Biol.

[197] Soneson C, Delorenzi M (2013). A comparison of methods for differential expression analysis of RNA-seq data. BMC Bioinf.

[198] Maza E (2016). In papyro comparison of TMM (edgeR), RLE (DESeq2), and MRN normalization methods for a simple two-conditions-without-replicates RNA-Seq experimental design. Front Genet..

[199] Benjamini Y, Hochberg Y (1995). Controlling the false discovery rate: a practical and powerful approach to multiple testing. J Roy Stat Soc.

[200] Liu Y, Beyer A, Aebersold R (2016). On the dependency of cellular protein levels on mRNA abundance. Cell.

[201] Gaudet P, Dessimoz C (2017). Gene ontology: pitfalls, biases, and remedies. Methods Mol Biol.

[202] Castro Dias M, Coisne C, Baden P, Enzmann G, Garrett L, Becker L (2019). Claudin-12 is not required for blood–brain barrier tight junction function. Fluids Barriers CNS..

[203] Fang Z, Cui X (2011). Design and validation issues in RNA-seq experiments. Brief Bioinform.

[204] Zhang Y, Chen K, Sloan SA, Bennett ML, Scholze AR, O’Keeffe S (2014). An RNA-sequencing transcriptome and splicing database of glia, neurons, and vascular cells of the cerebral cortex. J Neurosci.

[205] Zhang Y, Sloan SA, Clarke LE, Caneda C, Plaza CA, Blumenthal PD (2016). Purification and characterization of progenitor and mature human astrocytes reveals transcriptional and functional differences with mouse. Neuron.

[206] Zeisel A, Muñoz-Manchado AB, Codeluppi S, Lönnerberg P, La Manno G, Juréus A (2015). Cell types in the mouse cortex and hippocampus revealed by single-cell RNA-seq. Science.

[207] Ståhl PL, Salmén F, Vickovic S, Lundmark A, Navarro JF, Magnusson J (2016). Visualization and analysis of gene expression in tissue sections by spatial transcriptomics. Science.

[208] Maniatis S, Äijö T, Vickovic S, Braine C, Kang K, Mollbrink A (2019). Spatiotemporal dynamics of molecular pathology in amyotrophic lateral sclerosis. Science.

